# Direct Comparison between Förster Resonance Energy Transfer and Light-Induced Triplet–Triplet Electron Resonance Spectroscopy

**DOI:** 10.1021/jacs.3c04685

**Published:** 2023-10-15

**Authors:** Arnau Bertran, Laura Morbiato, Jack Sawyer, Chiara Dalla Torre, Derren J. Heyes, Sam Hay, Christiane R. Timmel, Marilena Di Valentin, Marta De Zotti, Alice M. Bowen

**Affiliations:** † Centre for Advanced Electron Spin Resonance and Inorganic Chemistry Laboratory, Department of Chemistry, University of Oxford, Oxford OX1 3QR, United Kingdom; ‡ Department of Chemical Sciences, University of Padova, 35131 Padova, Italy; § The National Research Facility for Electron Paramagnetic Resonance, Department of Chemistry, Manchester Institute of Biotechnology and Photon Science Institute, The University of Manchester, Oxford Road, Manchester M13 9PL, United Kingdom; ∥ Centro Interdipartimentale di Ricerca “Centro Studi di Economia e Tecnica dell’energia Giorgio Levi Cases”, 35131 Padova, Italy

## Abstract

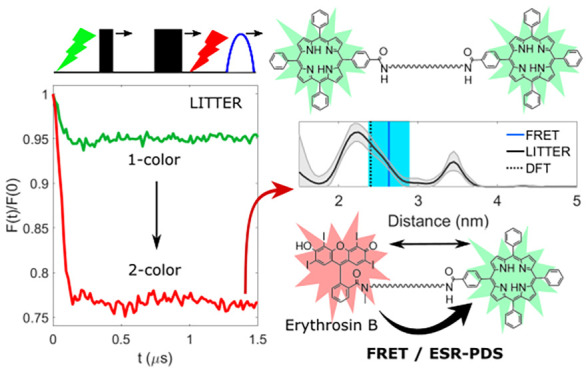

To carry out reliable and comprehensive structural investigations, the exploitation of different complementary techniques is required. Here, we report that dual triplet-spin/fluorescent labels enable the first parallel distance measurements by electron spin resonance (ESR) and Förster resonance energy transfer (FRET) on exactly the same molecules with orthogonal chromophores, allowing for direct comparison. An improved light-induced triplet–triplet electron resonance method with 2-color excitation is used, improving the signal-to-noise ratio of the data and yielding a distance distribution that provides greater insight than the single distance resulting from FRET.

Comparison between different methods in structural biology can be important to identify structural changes or prevent the interpretation of structural artifacts related to sample conditions or preparation. Electron spin resonance (ESR) pulsed dipolar spectroscopy (PDS) is a set of techniques for the study of conformational flexibility and disorder in complex biological macromolecular systems.^1−4^ Microwave (MW) pulses are used to measure the electron–electron magnetic dipolar interaction between two paramagnetic centers. PDS encompasses a range of techniques including double electron–electron resonance (DEER),^5−7^ relaxation induced dipolar modulation enhancement,^8,9^ double quantum coherence,^10^ and the single frequency technique for refocusing interactions^11^ that allow distance distributions between spin-bearing moieties to be measured from modulation of the signal intensity,^12^ giving direct information on the structure of the system.^13^ DEER, with nitroxide spin labels, is the most commonly used,^2^ accessing distances between 1.4 and 8 nm,^2,14^ sometimes extendable up to 14 nm.^15−17^ A similar range is accessible by Förster resonance energy transfer (FRET).^18,19^ Parallel DEER and FRET studies, including single-molecule FRET, have been performed on both chemical model systems and protein complexes; however, different labels are required, and direct distance comparison was not possible.^20−24^ Examples of dual spin-fluorescence labels have previously been reported using nitroxides chemically linked to fluorescent species; however, the presence of the nitroxide usually quenches the fluorescence, and it is necessary to chemically silence the nitroxide, e.g., by transforming the N–O^•^ radical into a N-OMe moiety to restore the fluorescence.^25−28^ While such species could be used for parallel DEER and FRET studies, the chemical modification required and the fact that the nitroxide is often not located close to the center of the fluorescent moiety introduce both additional synthetic steps and uncertainty in comparison with distances measured. Additionally, the nitroxide spin density, responsible for the dipolar interaction in PDS EPR methods, is shifted in space with respect to the excited state transition dipole moment, used in FRET; hence, distances measured by the two techniques cannot be expected to be the same in those cases, a previous caveat that the presented study overcomes.

Light-induced PDS methods have been developed based on the photogenerated triplet state of a 5(4′-carboxyphenyl)-*10,15,20-*triphenylporphyrin (TPP) moiety as photoswitchable spin-label,^29−31^ allowing measurement of its dipolar interaction with permanent paramagnetic centers, e.g., nitroxide, in synthetic model peptides^29,30,32−36^ and heme-containing proteins.^33,37−39^ The triplet can be used for detection and the permanent paramagnetic center pumped, as in light induced DEER,^29^ or the triplet state generated during the pulse sequence and the permanent paramagnetic center detected, as in laser-induced magnetic dipole spectroscopy.^33^ Subsequent studies replaced TPP with other chromophores.^40−44^ The photogenerated triplet is advantageous over permanent paramagnetic centers due to its strong spin polarization.^45^ Distances up to ca. 8 nm have been measured for nitroxide and TPP-containing systems,^32^ and orientation information can be extracted.^31,35^

The light-induced triplet–triplet electron resonance (LITTER) technique (Figure 1a)^46^ enables the measurement of the dipolar interaction between two photogenerated triplets in chromophore-containing molecules, which are ESR silent in their ground state, eliminating the need for permanent spin centers, as usually required for PDS including the use of metal ions.^47−50^ Electron spin-echo detection is performed on a triplet formed by a laser pulse preceding the MW pulse sequence, while the second “pump” triplet is generated by a time-variant laser pulse, changing the dipolar interaction between the two paramagnetic centers.^33^ The laser pump pulse, depolarized due to the use of a fiber optic in the light path, initiates the formation of an excited singlet state, which undergoes intersystem crossing to a triplet state. Formation of the T_+_ or T_–_ states results in a change of Ms, ΔMs = ±1, equivalent to the application of a microwave π-pulse, but exciting all orientations of the chromophore. Indeed, a MW pulse will only have a limited bandwidth on the order of MHz, exciting only a small portion of the triplet EPR spectrum, corresponding to a limited range of orientations of the triplet chromophore with respect to the external magnetic field. Conversely, the unpolarized laser pump pulse in LITTER, exciting all orientations of the chromophore, provides an effectively unlimited excitation bandwidth of the EPR triplet spectrum compared to MW pulses. The simple Hahn-echo detection sequence used for LITTER yields maximum signal intensity without the dead time associated with Hahn-echo detection 3-pulse DEER^5−7^ or 4-pulse relaxation induced dipolar modulation enhancement (RIDME),^8,9^ as the pumping laser pulse can occur concurrently with MW pulses and detected spin echo. The fixed position of all of the MW pulses means that the LITTER experiment is not sensitive to electron spin echo envelope modulations (ESEEM); therefore, no τ-averaging procedures are required unlike other PDS methods,^2,51^ and no complex phase cycles are required to remove unwanted echoes.^52^ The non-Boltzmann population of the triplet state enhances the signal intensity compared to permanent paragenetic centers rendering the LITTER method frequency independent as no gains are made from larger population differences at higher field/frequency,^53^ and the spectral width of the spectrum is controlled by the anisotropy of the zero-field splitting tensor.^54^

**Figure 1:**
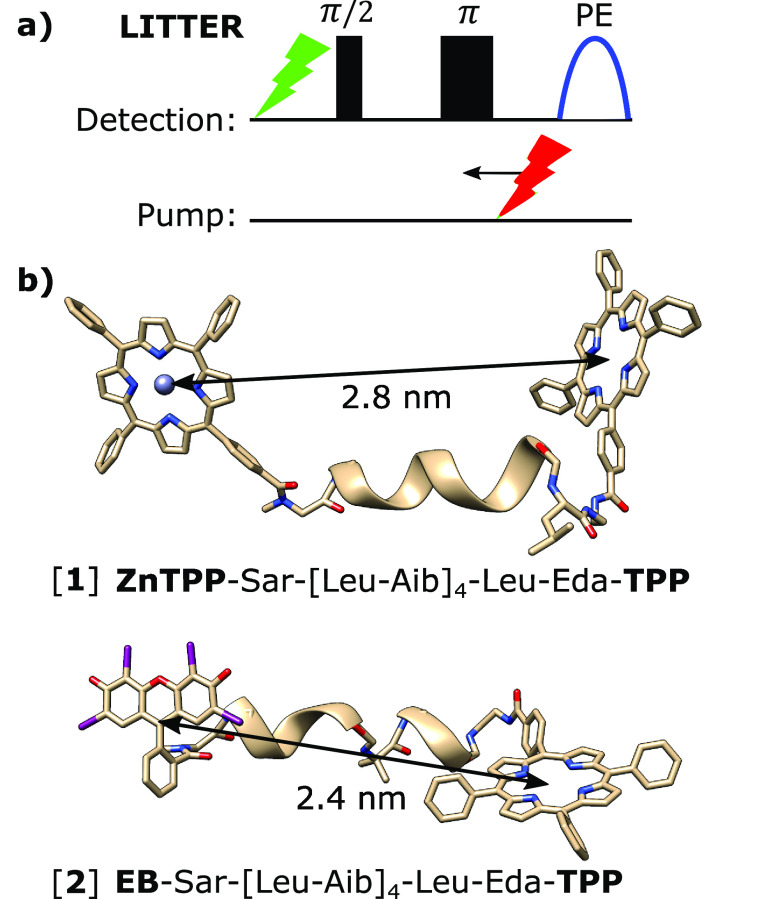
(a) LITTER pulse sequence: π/2 and π MW detection pulses (black) preceded by laser 1 (green) form a primary spin-echo (PE). The intensity is modulated by the formation of the second triplet by time-variant laser 2 (pump, red). (b) Amino acid sequences of [**1**] and [**2**], indicating the chromophore center-to-center distances determined by *in vacuo* DFT optimization (Figure S15) and chemical structures. Aib, α-aminoisobutyric acid; Eda, ethylenediamine. Other DFT optimized structures yielding higher energy local minima are presented in Figures S16 and S17 and Table S4.

In LITTER using homogeneous chromophores, the lack of photoexcitation selectivity limited the modulation depth (Section S2.5) and the dipolar modulation-to-noise ratio (MNR, Section S1.3).^46^

Here, we demonstrate that LITTER enables the first direct comparison between ESR PDS and FRET distance measurements using the same labels acting as both triplet spin centers and fluorophores. We show that LITTER can be performed using commercially available, porphyrin and nonporphyrin chromophores.^55^

Bis-labeled model peptides [**1**] and [**2**] containing orthogonal chromophore pairs, TPP with Zn(II) TPP (ZnTPP) and TPP with erythrosine B (EB), respectively, were synthesized to have a rigid α-helical structure resulting from alternating l-leucine−α-aminoisobutyric acid (Leu-Aib) residues (Figures 1b and S15). A sarcosine (Sar) linker was used to attach the labels to the N-terminus, avoiding the formation of colorless EB spirolactam.^31,43^ This is advantageous over previous strategies as it uses the more affordable nonderivatized chromophore and introduces a shorter linker.^42^ Zn(II) complexation was achieved directly with resin-bound TPP peptide, reducing the number of purification steps.^56^

A 2-color version of LITTER is shown in Figure 1a. Due to the longer *T*_m_ of the TPP triplet formed by photoexcitation at 512 nm (Figures 2a and 3a) at the measurement temperature compared to the triplets of EB and ZnTPP (Figures S11 and S14b), this signal was used for detection. The selective formation of triplets at 512 nm was studied by transient ESR (trESR) in 1:1 molar mixtures of free chromophores (Figure S12) and in [**1**] and [**2**] (Figure S13). This showed enhanced TPP triplet formation in labeled peptides; 65% TPP triplet signal was observed with a 35% EB or ZnTPP triplet signal for pairs of free chromophores. In [**1**] and [**2**], the proportion of TPP triplet signal increased to 85% and 100%, respectively. This suggests that energy transfer between the photoexcited ZnTPP or EB and the TPP quenches their excited states such that there is less or no observable signal from the triplet states of these moieties after excitation by laser 1 compared to the mixtures of free dyes. Laser 2 was set to 556 and 532 nm for [**1**] and [**2**], respectively, close to the maximum absorption of the pump chromophore (ZnTPP and EB) and at an absorption minimum of TPP (Figures 2a and 3a). Experimentally, it was determined that sample concentrations of 40 and 12 μM for [**1**] and [**2**], respectively, were optimal, giving an absorbance of approximately 0.7 at the second wavelength of the 2-color experiment, avoiding excessive attenuation of laser 2. A polynomial background correction was applied on LITTER data sets. The effect of different forms of background correction is explored in Section S2.4. The background form of LITTER includes contributions from both intermolecular dipolar interactions, as seen in the DEER experiment,^31^ and relaxation, as seen in RIDME,^57^ since the number of spin active species in the sample changes when laser 2 generates triplets.

**Figure 2:**
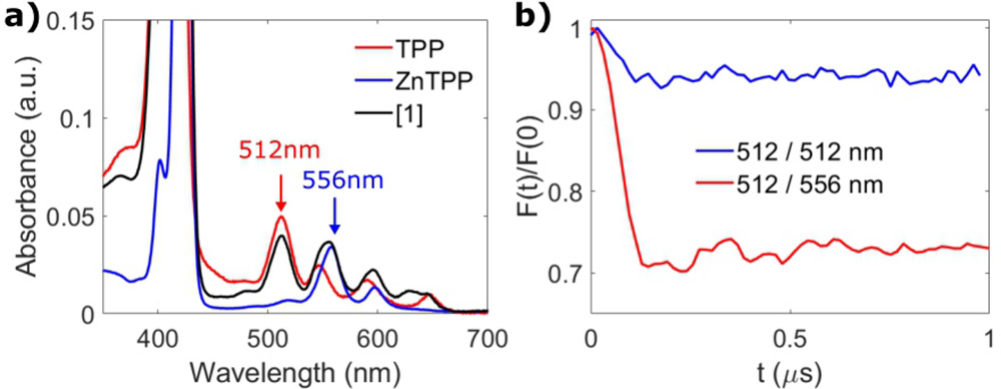
(a) Room temperature optical absorption spectrum of [**1**] (black), with reference spectra of TPP (red) and ZnTPP (blue), normalized to the maximum of the Soret band. (b) Background-corrected and normalized 1-color (512/512 nm, τ = 960 ns, scans = 200, blue) and 2-color (512/556 nm, τ = 1730 ns, scans = 7580, red) LITTER traces of 40 μM [**1**] with modulation depths of 5% and 27%, acquired on the most intense feature of the TPP triplet spectrum and not orientationally averaged. The modulation-to-noise ratio (MNR) corrected for the number of scans for the two experiments is red:blue = 0.75:0.50.

**Figure 3:**
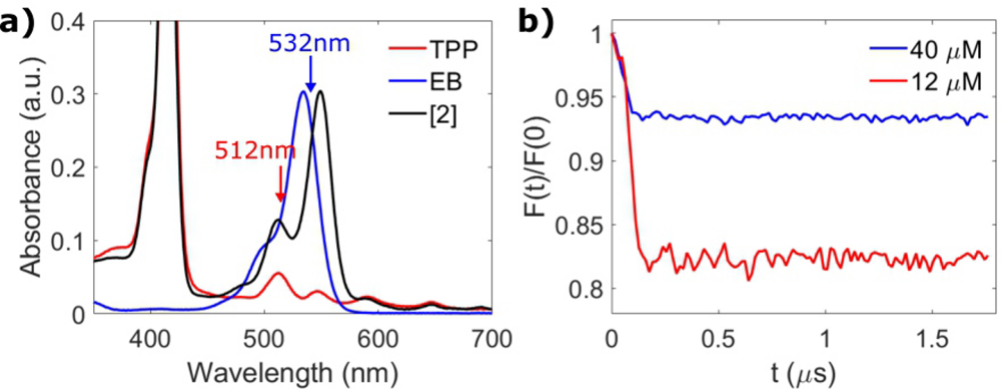
(a) Room temperature absorption spectrum of [**2**] (black), TPP (red), and EB (blue). (b) Background-corrected and normalized 2-color (512/532 nm) LITTER traces at 40 μM (τ = 1730 ns, scans = 7410, blue) and 12 μM (τ = 1730 ns, scans = 4820, red) [**2**] with modulation depths of 8% and 23%, acquired on the most intense feature of the TPP triplet spectrum and not orientationally averaged. The modulation-to-noise ratio (MNR) corrected for the number of scans for the two experiments is red:blue = 0.55:0.40.

The 2-color LITTER trace (512/556 nm) for 40 μM [**1**] yielded a modulation depth of 27% (Figure 2b), an approximately 2-fold improvement in modulation depth compared to previous 1-color LITTER experiments measured on bis-porphyrin model systems.^46^ For experiments measured on samples at the same concentration yielding similar detection echo intensities, this would result in a 4-fold reduction in acquisition time to yield the same MNR. This improvement is a direct result of better triplet formation selectivity in the 2-color experiment. When the experiment was repeated at 40 μM [**1**] with both lasers at 512 nm (512/512 nm), the modulation depth was reduced to 5% (Figure 2b) due to the low absorbance of ZnTPP at 512 nm (Figure 2a), demonstrating the importance of careful selection of the excitation wavelength of the LITTER experiment.

To investigate the suitability of nonporphyrin chromophores for LITTER, the 2-color experiment was repeated with 12 μM [**2**] using 512/532 nm for lasers 1 and 2, respectively. A maximum modulation depth of 23% was observed (Figure 3b) with a MNR similar to that obtained with [**1**] (Section S1.3). The smaller modulation observed for [**2**] compared to [**1**] may be attributed to a higher energy-transfer efficiency in the EB/TPP pair, which may diminish the formation of the EB triplet and the pump chromophore in the LITTER experiment, reducing the modulation depth. Evidence of a higher energy-transfer efficiency is seen in the results of the FRET experiments, where EB is the donor, and the degree of EB fluorescence quenching is significant. In the FRET experiment on system [**1**], TPP is the donor, and in the LITTER experiment, the TPP triplet is used for detection. Therefore, in system [**1**], a reduction in the number of TPP triplets due to resonant energy transfer from the excited singlet state would lead to a reduction in the detected echo intensity in the LITTER experiment rather than a reduction in the modulation depth. A hypothetical pair of labels where the quenching of the donor chromophore fluorescence is complete would likely not be suitable for the LITTER experiment, as there would be either no modulation or no signal to detect.

The large modulation depths obtained with 2-color LITTER constitute a significant improvement on previous 1-color experiments.^46^ However, energy transfer between chromophores quenches the excited state population, as observed by relative loss of the EB and ZnTPP triplet signal in trESR experiments (Figures S12 and S13). This prevents idealized modulation depth values from being realized experimentally (Section S2.5). According to our analytical expression (eq 1 in the Supporting Information; see Section S2.5 for derivation), the modulation depth of an ideal LITTER experiment is proportional to the efficiency of pump triplet formation by the second laser, and it decreases with increasing efficiency of pump triplet formation by the first laser. For the optimal 2-color experiments on [**1**] and [**2**], the maximum achievable modulation depths in the absence of energy transfer are predicted to be 0.60 and 0.55, respectively.

The singlet–singlet energy transfer and fluorescence properties of the chromophore pairs chosen for this study also allow for FRET measurements, and these were performed on both systems with 510 nm excitation and fluorescence detection between 525 and 750 nm. In [**1**], TPP acts as the donor and ZnTPP acts as the acceptor due to the higher absorbance of TPP at 510 nm. The relatively low fluorescence intensity (brightness) of TPP at 561 nm, the wavelength used for FRET analysis, meant that drift from the baseline comprised a non-negligible signal intensity (ca. 8%) and consequently a background correction was required for the determination of the distance (Section S2.8). The form of this background correction provides the main source of error in the interchromophore distance for [**1**]. Reported values of the quantum fluorescence yields (Φ_fl_) for unbound TPP are highly consistent in the literature at 0.13.^61^ The fluorescence quantum yield of TPP bound to a peptide (system [**3**], see Table S1 for the peptide sequence) was calculated relative to the TPP standard at 0.128 (see Sections S1.5 and S2.6 for details). Consequently, this value was used in the calculation of distances from the FRET data for system [**1**]. In Figure 4e, the dark blue region represents the possible interchromophore distances using different background corrections and the cyan region represents an error of ±5% in the calculation of R_0_ using the approximation κ^2^ = 2/3 for a system with low fluorescence anisotropy (see Sections S1.6 and S2.7).^62^

**Figure 4:**
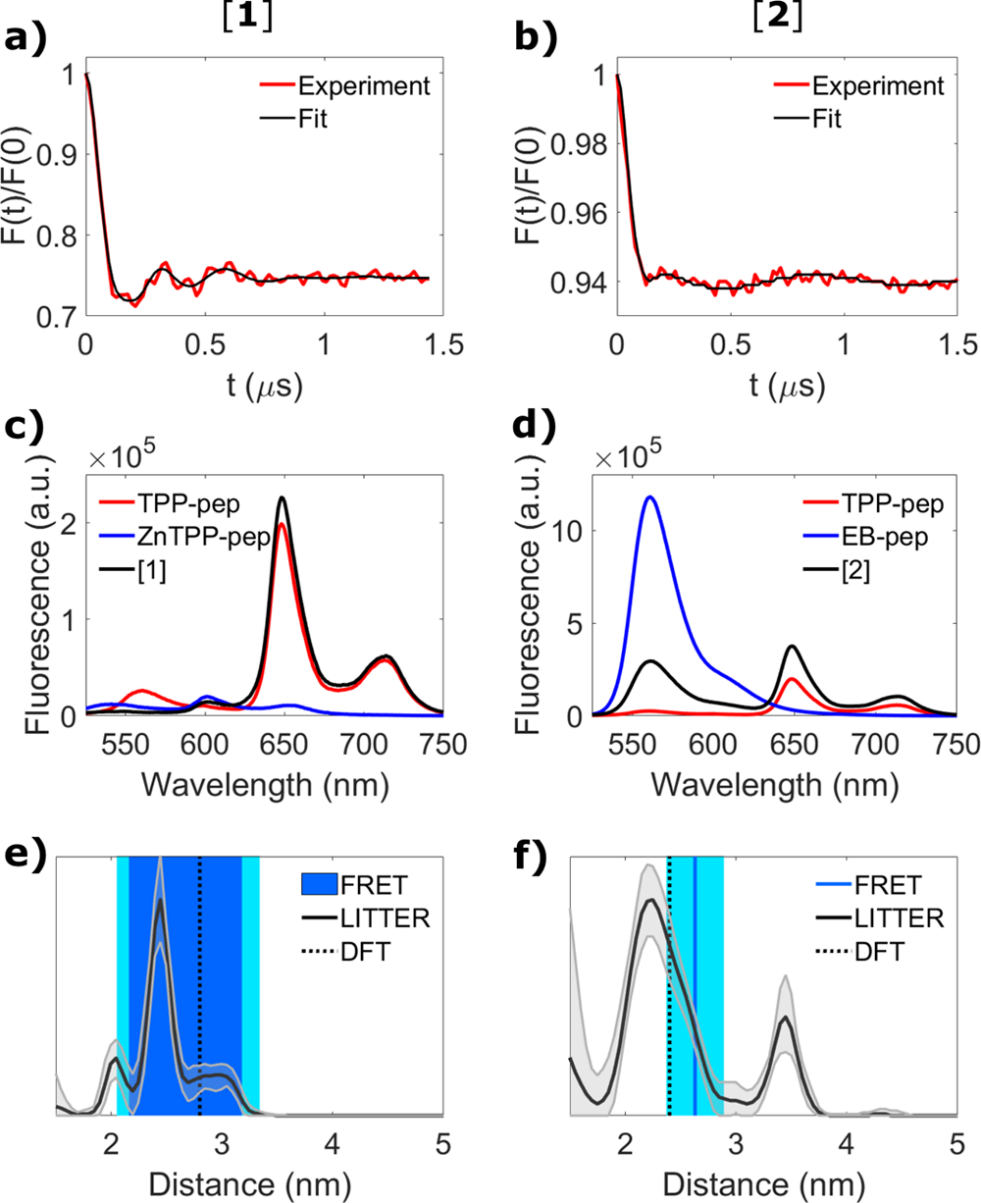
(a, b) 2-color LITTER traces averaged in orientation, background corrected, and normalized of [**1**] (a) (512/556 nm) and [**2**] (b) (512/532 nm) at 40 μM (red) with fits obtained using the Comparative DEER Analyzer in DEERAnalysis2022^58−60^ (black) with regularization parameter α of 0.22 and 0.31, respectively. (c, d) Concentration-normalized fluorescence spectra of [**1**] (c) and [**2**] (d) (black) and of singly labeled TPP peptide [**3**] (red), ZnTPP peptide [**4**] (c, blue), and EB peptide [**5**] (d, blue); see Table S1 for peptide sequences of [**3**], [**4**], and [**5**]. (e, f) Comparison of the distances determined for [**1**] (e) and [**2**] (f) by FRET (blue) with error bounds (cyan), LITTER (solid black line) with 95% confidence intervals (gray), which include analysis of the uncertainty in the background correction, and DFT (dotted black line).

TPP behaves as the FRET acceptor in [**2**] (Figure 4d) and EB is the donor, as EB exhibits higher absorption at 510 nm. The observed intensity for fluorescence emission was significantly higher for system [**2**] compared to system [**1**], and consequently, uncertainty in baseline drift has a much smaller effect on the data recorded for system [**2**], ca. 0.1% of the signal compared to 8% of the signal for system [**1**]. This indicates that the TPP–EB pair is better suited to FRET measurements than the TPP–ZnTPP in terms of the achievable signal-to-noise ratio of the fluorescence emission spectra. Reported values of Φ_fl_ for EB vary between 0.02 for the free dye and 0.17 for EB adsorbed onto a protein surface.^63^ Consequently, it was necessary to determine the fluorescence quantum yield for the EB dye attached to a peptide (system [**5**]; see Table S1 for peptide sequence), and this was achieved by comparison to a Rhodamine 6G standard and yielded a value of Φ_fl_ = 0.095 (see Sections S1.5 and S2.6 for details). This value was used in the determination of the inter-chromophore distance by FRET for [**2**]. The determined FRET distance is shown as a dark blue line in Figure 4f. A cyan region of uncertainty, ±10% of the calculated value, is also depicted to reflect the use of the κ^2^ = 2/3 approximation on a system with higher fluorescence anisotropy.^64^

This work shows that FRET distances can be directly compared to LITTER distance distributions using dual spin/fluorescent labels, which was impossible in conventional DEER.^20−23^ Spin–spin distance distributions were extracted using orientationally averaged LITTER traces and analyzed by a Comparative DEER analyzer in DEERAnaylsis2022 with an orientation-independent kernel^58−60^ (Figure 4, Section S2.4, Figure S16). The main features of the LITTER distance distributions are consistent with the density functional theory (DFT) optimized geometries of [**1**] and [**2**] (Figure S15). The distribution widths suggest a significant degree of conformational flexibility in solution, which could originate from different conformations of the chromophores with respect to the α-helix; a second conformation of [**2**] could be responsible for the peak at 3.5 nm. Good agreement is seen with the FRET-determined distance ranges (Figure 4e,f). Ensemble FRET is not able to determine different populations; a second population with a longer interchromophore distance in [**2**] would explain the offset of the FRET distance range from the main feature seen in the LITTER distribution. Distance distributions could be determined using single-molecule FRET provided that a suitably large number of molecules were analyzed.^22^

In conclusion, we have reported the first LITTER experiments with optically orthogonal chromophores and 2-color photoexcitation, obtaining larger modulation depths corresponding to a 4-fold shortening of the acquisition time with respect to previous 1-color measurements. LITTER can also exploit nonporphyrin chromophores including some commonly used as biological labels; examples include EB^43^ and other fluorophores used in light induced PDS methods such as ATTO Thio 12, Rose Bengal, Eyosin Y, and C_60_.^31,41,42,65,66^ This makes the technique directly transferable to structural biology applications. Our dual spin/fluorescent labels enabled the first parallel distance measurements by ESR and FRET on exactly the same molecules, allowing for direct comparison and exploiting the complementarity of the two techniques, with LITTER yielding distance distributions and FRET providing information in the room-temperature liquid state.

The 2-color LITTER represents a dramatic step forward in biological structural determination in systems where FRET labels can be added or innate chromophores used. The choice of orthogonal chromophores provides a significant improvement in data quality compared to the initial homo-LITTER experiments. Furthermore, the selective optical addressability of each chromophore has the potential to enable unambiguous determination of the PDS distance in systems with more than two labels, without the interference of multispin effects.^67,68^ Optical orthogonality is easier to achieve and allows a wider choice of labels than spin-label orthogonality.^69−71^ Therefore, 2-color LITTER offers a promising route to controlling complexity in PDS of multispin systems. There is also the potential for increasing the modulation depth by further reducing the spectral overlap and unwanted excitations by choosing different chromophore pairs and excitation wavelengths.

LITTER requires low concentrations and is likely suitable for in-cell ESR studies, where conventional nitroxide labels degrade rapidly.^72,73^ When combined with fluorescence microscopy, LITTER could provide location-specific structural information for labeled biomolecules inside cells.

## Data Availability

The raw data and processing information are available online under the following DOIs: README ESR file 10.48420/22177202, trESR data 10.48420/22177199, LITTER data 10.48420/22177196, and FRET data 10.48420/21961571.

## Supporting Information Available

Methods: synthesis, sample preparation, ESR spectroscopy, FRET, quantum yield measurements, fluorescence anisotropy, and DFT calculations; results: purification and analysis, characterization of free chromophores, characterization of [**1**] and [**2**], LITTER analysis, LITTER modulation depth prediction, and quantum yield measurement, fluorescence anisotropy, and FRET analyses (PDF)

## Notes

For the purpose of open access, the author has applied a Creative Commons Attribution (CC BY) license (where permitted by UKRI, the ‘Open Government License’ or ‘Creative Commons Attribution No-derivatives’ (CC BY-ND) license may be stated instead) to any Author Accepted Manuscript version arising.

## Abbreviations

ESEEM: electron spin echo envelope modulation
ESR: electron spin resonance
PDS: pulsed dipolar spectroscopy
MW: microwave
DEER: double electron–electron resonance
FRET: Förster resonance energy transfer
LITTER: light-induced triplet–triplet electron resonance
MNR: modulation-to-noise ratio
ZnTPP: TPP with Zn(II)
EB: erythrosin B
RIDME: relaxation induced dipolar modulation enhancement
trESR: transient ESR
TPP: 5(4′-carboxyphenyl)-*10,15,20*-triphenylporphyrin
DFT: density functional theory
