# Supplementary material for: Direct Comparison between Förster Resonance Energy Transfer and Light-Induced Triplet–Triplet Electron Resonance Spectroscopy
Source: J Am Chem Soc. 2023 Oct 15;145(42):22859–65. doi: 10.1021/jacs.3c04685 (PMC10603778; doi:10.1021/jacs.3c04685)
Supplement: Supplementary file 1 — ja3c04685_si_001.pdf [file ja3c04685_si_001.pdf]

Supporting Information for

Direct comparison between Förster Resonance Energy Transfer and Light-Induced Triplet–Triplet Electron Resonance Spectroscopy

*Arnau Bertran [1], Laura Morbiato [2⁋], Jack Sawyer [3⁋], Chiara Dalla Torre [2⁋], Derren J. Heyes[3], Sam Hay[3], Christiane R. Timmel [1], Marilena Di Valentin [2,4], Marta De Zotti[*2,4] and Alice M. Bowen[*3]*

[1] Centre for Advanced Electron Spin Resonance and Inorganic Chemistry Laboratory, Department of Chemistry, University of Oxford, Oxford OX1 3QR, United Kingdom.

[2] Department of Chemical Sciences, University of Padova, 35131 Padova, Italy.

[3] The National Research Facility for Electron Paramagnetic Resonance, Department of Chemistry and Photon Science Institute, The University of Manchester, Oxford Road, Manchester M13 9PL, United Kingdom.

[4] Centro Interdipartimentale di Ricerca "Centro Studi di Economia e Tecnica dell'energia Giorgio Levi Cases", 35131 Padova, Italy.

⁋Authors contributed equally.

*Corresponding authors: alice.bowen@manchester.ac.uk, marta.dezotti@unipd.it

CONTENTS

S1. Methods

S1.1. Synthesis

S1.2. Sample preparation

S1.3. ESR spectroscopy

S1.4. FRET

S1.5 Quantum Yield Measurements

S1.6 Fluorescence Anisotropy

S1.7. DFT calculations

S2. Results

S2.1. Purification and analysis

S2.2. Characterization of the free chromophores

S2.3. Characterization of [**1**] and [**2**]

S2.4. LITTER analysis

S2.5. LITTER modulation depth prediction

S2.6 Quantum Yield Measurements analysis

S2.7 Fluorescence Anisotropy analysis

S2.8. FRET analysis

S1. EXPERIMENTAL METHODS

S1.1. Synthesis

Fluorenylmethyloxycarbonyl(Fmoc)-protected amino acids, active agents and solvents for peptide synthesis were purchased from Sigma-Aldrich. Fmoc-1,4-*trans*-aminocyclohexylcarboxylic acid (Fmoc-CHX), Ethyl cyano(hydroxyimino)acetate (Oxyma Pure) and diisopropylcarbodiimide (DIC), were acquired from Iris Biotech. All peptides (Table S1) except from [**3**] were obtained by manual Solid Phase Peptide Synthesis (SPPS) on a 1,2-diaminoethane-trityl resin (Iris Biotech, Marktredwitz, Germany). Fmoc-deprotection was achieved by repeated treatment with 20% piperidine solution in dimethylformamide (DMF) (5 and 10 min, respectively). The 1-hour coupling steps were generally carried out twice, since they all involved the poorly reactive Aib residue. Oxyma Pure and DIC were used as activating agents. All the coupling steps were carried out with three equivalent excess of the activated residue. Finally, coupling reactions repeated three times (1h, 1h, and overnight, respectively) with three equivalents of the chosen chromophore [5-(4-Carboxyphenyl)-10,15,20-(triphenyl)porphyrin (TPP-COOH) or Erythrosin B (EB)], DIC and Oxyma Pure afforded the N-terminal capping quantitatively.

Analysis by ESI-HRMS of a small cleavage just after TPP coupling showed the presence of a small amount of a byproduct the molecular weight of which is compatible with Ac-peptide (instead of the TPP-peptide). We performed the TPP coupling under a variety of experimental conditions, modifying either active agents or reaction times but could not avoid its formation. It was easily removed by flash chromatography, after Zn insertion and peptide cleavage from the resin. Zn complexation onto the TPP-peptide to make [**4**] was obtained directly on resin, by several treatments with a saturated solution of $Zn(Ac)_2$.[1] Briefly, Zinc Acetate was dissolved in a

DMF/Ethanol mixture 1:1. Quantitative complexation on resin required two overnight treatments with fresh solutions of $Zn(Ac)_2$ (Zinc Acetate, 50 equiv.).

Peptide cleavage from the resin was achieved by three treatments (two 1-hour reaction and one overnight) with 30% 1,1,1,3,3,3-hexafluoro*iso*propanol (HFIP) in dichloromethane (DCM), as previously described.[2] The filtrates were collected and concentrated under a flow of N_2. The crude singly-labelled compounds [**4**] and [**5**] were purified by flash chromatography [gradient elution: from 99:1 to 74:26 DCM/CH_3OH (MeOH)] to remove traces of impurities and then the respective bis-labelled compounds [**1**] and [**2**] were synthesized by reaction with 0.9-1.1 equivalents of TPP-COOH in anhydrous DCM, using 1-ethyl-3-(3-dimethylaminopropyl)carbodiimide (EDC) and 1-hydroxy-7-azabenzotriazole (HOAt) as coupling reagents, in the presence of N-methylmorpholine (NMM) to pH 8. The final products were purified to >90% (apart from [**4**], which reached >80%) by flash chromatography using Silica gel (60 Å, 70-230 mesh, 63-200 μm – Sigma-Aldrich), at the following elution conditions: [**1**], DCM/MeOH 96:4; [**2**]: from 99:1 to 90:10 DCM/MeOH.

[**3**] was synthesized on the semiautomatic peptide synthesizer Biotage MultiSynTech, using Oxyma pure and DIC as active agents. We used the 2-Chlorotrityl chloride resin with an experimentally calculated loading of 0.84 mmol/g and DMF as solvent for all couplings except the first one. To link the first amino acid (Fmoc-Chx) and calculate the loading we swelled the resin 5 min in DCM and then we added 1 eq. of Fmoc-Chx, 2.5 eq. of N,N-Diisopropylethylamine (DIPEA) in DCM. After 60 min, we washed the resin twice with DMF and then perform a capping with DCM/MeOH/DIPEA 16:3:1 mixture. We then washed the resin with DMF and remove the Fmoc protecting group with 20% solution of Piperidine in DMF, collecting and analyzing the waste by UV to determine the final loading of 0.84 mmol/g. We used 3 equivalents of the incoming, activated amino acid residue for each coupling, and performed all the coupling twice,

because of the presence of the poorly reactive, tetrasubstituted, non-proteinogenic amino acid Aib. Once the sequence was completed, the resin was dried in DCM and cleaved for 1h using 30% TFA in DCM. The solvent was removed under N_2 flow and the peptide was precipitated in diethyl ether. After centrifugation, the pellet was dried under N_2 and the crude peptide was purified by means of medium-pressure chromatography on a Biotage Isolera Prime instrument and obtained with a purity higher than 95%.

Table S1. Peptide sequences synthesized and studied in this work.

Name	Sequence[a]
[**1**]	TPP(Zn)-Sar-Leu-Aib-Leu-Aib^5-Leu-Aib-Leu-Aib-Leu^{10}-NH-CH_2-CH_2-NH-TPP
[**2**]	EB-Sar-Leu-Aib-Leu-Aib^5-Leu-Aib-Leu-Aib-Leu^{10}-NH-CH_2-CH_2-NH-TPP
[**3**]	TPP-Sar-Leu-Aib-Leu-Aib^5-Leu-Aib-Leu-Aib-Leu^{10}-NH-Cyclohexyl-COOH
[**4**]	TPP(Zn)-Sar-Leu-Aib-Leu-Aib^5-Leu-Aib-Leu-Aib-Leu^{10}-NH-CH_2-CH_2-NH_2
[**5**]	EB-Sar-Leu-Aib-Leu-Aib^5-Leu-Aib-Leu-Aib-Leu^{10}-NH-CH_2-CH_2-NH_2

[a]TPP, 5-(4-Carboxyphenyl)-10,15,20-(triphenyl)porphyrin; EB, Erythrosin B; Aib, α-aminoisobutyric acid; Sar, sarcosine

Product characterization was achieved by reverse phase high pressure liquid chromatography RP-HPLC [Agilent 1200Series or GE Pharmacia instrument equipped with a DAD UV-detector and a binary elution system: A, H_2O/CH_3CN (9:1 *v*/*v*)+0.05%trifluoroacetic acid (TFA); B, CH_3CN/H_2O (9:1 *v*/*v*)+0.05%TFA] on a Phenomenex C4 column (30×250 mm, particle size: 5 μm, pore size: 300 Å) and by Electrospray ionization, high-resolution mass spectrometry (ESI-HRMS) on a Waters Xevo or Micromass Mass Spectrometer.

S1.2. Sample preparation

Samples for ESR were prepared at a molecular concentration of 40 μM for [**1**], 40 and 12 μM for [**2**], and 100 μM for the free chromophores, in d_6-ethanol, and were loaded in 4 mm outer-diameter quartz tubes to a sample height of 5 mm (40 μL). The solvent was chosen to improve solubility and favor helicity of the peptide backbones. Samples were degassed by several freeze-pump-thaw cycles and flash-frozen in liquid nitrogen prior to insertion into the spectrometer. The optimal samples of [**1**] and [**2**] for LITTER measurements had concentrations corresponding to an absorbance of 0.7 at wavelength of the second laser used in the 2-color experiment.

S1.3. ESR spectroscopy

ESR experiments were performed at X-band (microwave frequency = 9.7 GHz) and a temperature of 20 K in a pulsed spectrometer (ElexSys E680, Bruker) using a dielectric resonator (EN 4118XMD5, Bruker) and an Oxford Instruments cryostat. The two-laser setup was as previously reported.[3] Laser 1 (OPOlette, Lambda Photometrics) was directed into the resonator through the optical window of the cryostat, while laser 2 (VersaScan OPO, GWU) was delivered via a 1 mm x 10 m optical fiber (FT1000-EMT, Thorlabs) directly inserted inside the sample tube. Laser energies were between 2 mJ per flash for laser 1 and 3 mJ per flash for laser 2. For the pulsed experiments, a delay pulse generator was used to externally trigger both

lasers and the spectrometer at a repetition rate of 20 Hz, and the microwave pulses were moved forward in time with respect to the fixed laser flashes. All spectra were acquired with one shot per point.

Time-resolved ESR (trESR) was carried out in a critically-coupled resonator using Laser 1 only, without field modulation or phase sensitive detection. The signal was averaged around the intensity maximum of the each time resolved trace to yield a spectrum with intensity against field (Fig. S11). Spectra were simulated using the Matlab® *EasySpin* routine (*pepper* function).[4]

Light-induced pulsed ESR characterization (i.e. field sweeps, phase-memory time and delay-after-flash experiments) were performed in an over-coupled resonator using a standard Hahn-echo sequence preceded by a laser flash (laser 1 – DAF – π/2 – τ – π – τ – echo), with rectangular 16-32 ns π/2- π pulses and a τ value of 320 ns.

LITTER traces were acquired using the following pulse sequence: laser 1 – DAF – π/2 – τ – π – τ' – laser 2 – τ'' – echo, τ = τ' + τ'', with rectangular 16-32 ns π/2- π pulses. The delay between flashes was DAF = 7 μs and the time increment was 16 ns. Measurements were carried out at different values of the external magnetic field, and the exact τ delays were adjusted to match electron spin envelope modulation (ESEEM) maxima observed in the phase-memory-time experiments at the corresponding field positions (Fig. S14b), with τ values of 1730 and 1616 ns for the Y^- and Z^- traces, respectively. Unlike Double Electron-Electron Resonance no τ-averaging is needed in the LITTER experiment as there are no microwave pulses moving

relative to one another to cause ESEEM in the LITTER trace. Experimental parameters are presented in Table S2.

LITTER traces were phased and a third-order polynomial background correction was applied using the Matlab® program DEERAnalysis2022.[5] The effect of using other background corrections has been tested, see Section S2.4. The output files form the DEERAnalysis procedure using the third order polynomial correction are included in the data repository.

Modulation-to-noise ratios (MNR) were calculated dividing the experimental modulation depths by the noise intensities estimated as the root-mean-square deviations (RMSD) of the form factors after complete damping of the dipolar oscillations. The MNR increases with the square root of the number of scans and also has a dependence on the τ-value used due to ESEEM interactions and the phase memory decoherence, see Fig. S11. The MNR values for each dataset presented in the manuscript are given in Table S2. For direct comparison of MNR values it is ideal to compare traces recorded under conditions with the same τ-value and number of scans. In Fig. 2 we compare traces recorded for 2-colour (Fig. 2b red trace) and 1-colour (Fig. 2b blue trace) LITTER experiments on system [**1**] at the TPP triplet signal maximum (338.5 mT), a lower modulation depth is expected for the 1-colour experiment as excitation of the ZnTPP chromophore at 512nm is low. Normalizing the MNRs for these two experiments to the number of scans recorded by dividing by the square root for the number of scans gives a ratio of MNRs for 2-colour:1-colour of 0.75:0.5. Given than the 1-colour experiment on [**1**] was recorded with a shorter τ-value (τ = 960 ns) where the echo intensity if

higher due to less phase memory decoherence, compared to the 2-colour experiment (τ = 1760 ns), for experiments recoded with the same τ-value the relative MNR for the 2-experiment is expected to be relatively larger. A fairer comparison might be the 2-color LITTER recorded on [**1**] compared to a 1-color experiment recorded on a system with identical TPP chromophores, and also recorded on the signal maxima, reported previously.[3] In this case comparing experiments recoded with identical τ-values (τ = 1760 ns) and 4800 scans in both cases; for the 2-colour experiment recorded on [**1**], MNR = 47 for 2-color LITTER compared to MNR = 27 for the 1-color experiments recorded on a bis-TPP system. This gives a ratio of the MNR values for 2-color LITTER(ZnTPP-TPP):1-color LITTER(TPP-TPP) = 0.68:0.39.

For results recorded on system [**2**] at the signal maximum (338.5 mT) and different concentrations of 12 μM (Fig. 3b, red trace) and 40 μM (Fig. 3b, blue trace). Considering only the first 4800 scans recorded for each of these experiments the MNRs are: MNR = 38 at 12 μM *vs* MNR = 23 at 40 μM. This gives a ratio of the MNR values for 12 μM:40 μM = 0.55:0.33. Some variation in the increase of MNR with number of scans is expected as the power of the laser output reaching the sample can vary over time. In our experimental set up one source of this is the coupling of laser 2 into the fiber optic, which can deteriorate due to damage of the fiber optic tip. Cracking of the solvent glass can also affect the laser power reaching parts of the sample as light is scattered by the cracks. It is for this reason that the ratio considering all of the scans recorded and normalized for the number of scans is slightly different; 12 μM (red trace):40 μM (blue trace) = 0.55:0.40.

Recent work has shown signal-to-noise can be improved in Light Induced Pulsed Dipolar Spectroscopy methods by using Carr–Parcel–Meiboom–Gill (CPMG) blocks.[6] In the future such a method could also be applied to LITTER.

Table S2. Experimental details for each LITTER data sets presented, calculated MNR value for each dataset, normalized MNR (MNR√scans), and a link to the figure in the main paper in which the data is presented.

Molecule	Magnetic field (mT)	Detection frequency	Sample concentration	Excitation wavelengths probe (laser 1)/pump (laser 2)	τ	Scans	MNR for data recorded	MNR/ √scans	Figures in which data are presented
[1]	336.3	9.7 GHz	40 µM	512 / 556 nm	1730 ns	19880	19	0.13	Raw trace: Fig. S20b Included in orientation averaged trace in Fig. 4a
	388.5	9.7 GHz	40 µM	512 / 556 nm	1616 ns	6640	21	0.26	
	338.5	9.7 GHz	40 µM	512 / 556 nm	1730 ns	7580	65	0.75	Raw trace: Fig. S20b Included in orientation averaged trace in Fig. 4a. Red trace in Fig. 2b and Fig. S18
	338.5	9.7 GHz	40 µM	512 / 512 nm	960 ns	200	7	0.5	Blue trace in Fig. 2b and Fig. S18
[2]	336.3	9.7 GHz	40 µM	512 / 532 nm	1730 ns	15360	22	0.18	Raw trace: Fig. S20c Included in orientation averaged trace in Fig. 4b
	388.5	9.7 GHz	40 µM	512 / 532 nm	1616 ns	23200	24	0.16	
	338.5	9.7 GHz	40 µM	512 / 532 nm	1730 ns	7410	34	0.40	Raw trace: Fig. S20c Included in orientation averaged

									trace in Fig. 4b. Blue trace in Fig. 3b and Fig. S19
	338.5	9.7 GHz	12 μM	512 / 532 nm	1730 ns	4820	38	0.55	Red trace in Fig. 3b and Fig. S19

For the orientational averaging of the LITTER datasets, the dipolar traces acquired at different field positions (Fig. S20a-c) were averaged weighted by the corresponding spectral intensities prior to third-order polynomial background correction (Fig. S20d, e) performed in DeerAnalysis2022.[5] The resulting form factor (Fig. 4a, b) was then analyzed using the Comparative Deer Analyzer routine[7,8] to extract the corresponding distance distribution and confidence intervals (Fig. 4c, d). 2-color LITTER traces of [**2**] at different field positions had been acquired with the 40 μM sample (Fig. S20c) before carrying out the concentration-dependent study to maximize the modulation depth at 12 μM (Fig. 3b). For this reason, LITTER distance analysis of [**2**] was done on the 40 μM sample, with an averaged form factor with a modulation depth smaller than the maximum achieved at 12 μM (Fig. 4b). It was deemed not necessary to repeat this analysis at 12 μM, as the maximum modulation depth was already demonstrated with the single trace recorded at 12 μM (Fig. 3b) and no concentration-dependence of the distance distribution is expected in PDS at such low concentrations.

S1.4. FRET

Samples for FRET were prepared in ethanol (99.8% analytical reagent grade, Fisher Scientific). Initial stocks of 5 μM of each compound were prepared, from which dilutions gave samples with identical absorbance intensities at the excitation wavelength used. Final concentrations of 0.7μM for compounds [**1**], [**3**], and [**4**], 1 μM for compound [**2**], and 2 μM for compound [**5**]

were prepared. UV-Vis spectra for extinction coefficient and spectral overlap determination were acquired with a Cary 60 UV-Vis spectrometer (Agilent Technologies). Fluorescence emission spectra were recorded at room temperature on an FLS920 series spectrometer (Edinburgh Instruments) equipped with an Xe900 xenon arc lamp (Edinburgh Instruments). An excitation wavelength of 510 nm was used for both FRET pairs, recording emission scans from 525-750 nm in 1 nm steps, and a dwell time of 1 s. An excitation and emission bandwidth of 5 nm was used in all cases.

For each pair, the Förster distance (R_0) was calculated as

$$R_0 = \left(\frac{20.7\kappa^2\Phi_{\text{fl}}}{128\,\pi^5\,N_A\,n^4}J\right)^{\frac{1}{6}}$$

where κ is the dipole orientation factor (κ^2 taken to be 2/3), Φ_{fl} is the fluorescence quantum yield of the donor in the absence of acceptor, n is refractive index of medium, and J is the spectral overlap integral, calculated as

$$J = \int f_D(\lambda)\varepsilon_A(\lambda)\lambda^4\,d\lambda$$

where $f_D(\lambda)$ is the normalized donor emission spectrum and $\varepsilon_A(\lambda)$ is the normalized acceptor absorption spectrum. [9] The FRET efficiency (E) is calculated from the fluorescence quenching of the donor as follows:

$$E = 1 - \frac{F_{DA}}{F_D} \qquad \textbf{Equation S1}$$

where F_D is the fluorescence emission from the donor alone, and F_{DA} is the fluorescence emission from the donor in the presence of the acceptor. In all cases, F_D and F_{DA} were corrected for concentration and as described below, in some cases these fluorescence emission values were also corrected for background signal by subtracting the counts measured at the lowest wavelength of the emission scan.-The donor-to-acceptor distance (r) is calculated using the FRET efficiency as follows:[9]

$$r = R_0\left(\frac{1}{E-1}\right)^{\frac{1}{6}} \qquad \textbf{Equation S2}$$

S1.5. Quantum Yield Measurements

Quantum yields can be measured by measuring the integrated fluorescence intensity of an unknown system (I) and comparing this to the integrated fluorescence intensity to a known reference standard (I_R) that excites and emits at similar wavelengths to the unknown. It is important that the optical density of both the unknown (OD) and reference (OD_R) are measured and the refractive index of the medium for the unknown (n) and reference (n_R) are also known. The fluorescence quantum yield of the unknown (φ_{fl}) can then be calculated relative to the fluorescence quantum yield of the reference ($\varphi_{fl,R}$) using the equation:[9]

$$\varphi_{fl} = \varphi_{fl,R}\left(\frac{I}{I_R}\right)\left(\frac{OD}{OD_R}\right)\left(\frac{n^2}{n_R^2}\right) \qquad \textbf{Equation S3}$$

The fluorescence quantum yield, for EB-pep [**5**], has been determined for the given experimental conditions by comparison to rhodamine 6G (R6G). R6G has a well-established fluorescence quantum yield, known to be 0.95 ±0.005 in ethanol.[10] In the case of TPP-pep [**3**], free unbound TPP in ethanol was used as a reference, which has a well-known fluorescence quantum yield of 0.13.[11]

Samples of R6G, free unbound TPP, EB-pep [5] and TPP-pep [5] were prepared in ethanol (99.8% analytical reagent grade, Fisher Scientific). Initial stocks of 100 μM of each compound were prepared, from which dilutions gave samples with identical absorbance intensities at the excitation wavelength used (510 nm). UV-Vis spectra were acquired with a Cary 60 UV-Vis spectrometer (Agilent Technologies). Final concentrations of 0.25μM for R6G, 20 μM for compound [5] and 2 μM for compound [3] and free unbound TPP were prepared.

Fluorescence emission spectra were recorded for all samples under identical conditions: spectra acquired at room temperature on an FLS920 series spectrometer (Edinburgh Instruments) equipped with an Xe900 xenon arc lamp (Edinburgh Instruments). An excitation wavelength of 510 nm was used for all chromophores, recording emission scans from 525-800 nm in 1 nm steps, and a dwell time of 1 s. An excitation bandwidth of 1nm and emission bandwidth of 5 nm was used in all cases. Results are reported in Section S2.6

S1.6. Fluorescence Anisotropy

Steady state fluorescence anisotropy measurements were performed on all of the peptide samples [**1**]-[**5**]. Samples of each of [**1**]-[**5**] were prepared in ethanol (99.8% analytical reagent grade, Fisher Scientific). Compounds [**1**],[**2**], and [**4**] were 40 μM, 10 μM for [**5**], and 2 μM for [**3**]. Excitation emission spectra were recorded of each sample under identical conditions: spectra acquired at room temperature on an FLS920 series spectrometer (Edinburgh Instruments) equipped with an Xe900 xenon arc lamp (Edinburgh Instruments). Polarizers were positioned in the beam path both before (excitation polarizer) and after (emission polarizer) the sample cuvette. An excitation wavelength of 510 nm was used for all chromophores, recording emission scans from 525-750 nm in 1 nm steps, and a dwell time of 1 s. An excitation bandwidth of 1nm and emission bandwidth of 5 nm was used in all cases. Scans recorded for each sample in four polarization configurations with each polarizer either vertical or horizontal (V = vertical 0° polarizer rotation, H = horizontal 90° polarizer rotation), yielding four fluorescence spectra. The four spectra can be defined: I_{VV}, I_{VH}, I_{HH} and I_{HV}, where the first subscript corresponds to the polarization of the excitation beam and the second subscript to the emission polarization. In this way I_{VV} can also be defined as having parallel polarizations ($I_{||}$) and I_{VH} as having perpendicular polarization ($I_{\perp}$)

Steady state anisotropy for a given emission wavelength ($r(\lambda_{em})$) was determined by:[12]

$$r(\lambda_{em}) = \frac{GI_{VV}(\lambda_{em}) - I_{VH}(\lambda_{em})}{GI_{VV}(\lambda_{em}) + 2I_{VH}(\lambda_{em})} \qquad \textbf{Equation S4}$$

Where $G = I_{HH}(\lambda_{em})/I_{HV}(\lambda_{em})$, a factor to correct for the polarization dependence of the spectrometer. Results are reported in Section S2.7.

S1.7. DFT calculations

Initial molecular geometries were built using UCSF Chimera.[10] Geometry optimizations and spin density calculations were performed *in vacuo* using Gaussian® 16 (revision A.03).[11] Ground state geometry optimizations of [**1**] and [**2**] were carried out in the singlet state, using the PBE1PBE functional and the 6-31g(d) basis set and replacing ZnTPP by TPP in [**1**] and I by H in [**2**]. Geometry optimizations and spin density calculations of the free chromophores in their triplet state were performed using the functional B3LYP, with the basis sets Def2-SVP (for H, C, N and O) and Def2-TZVP (for Zn and I).

S2. RESULTS

S2.1. Purification and analysis

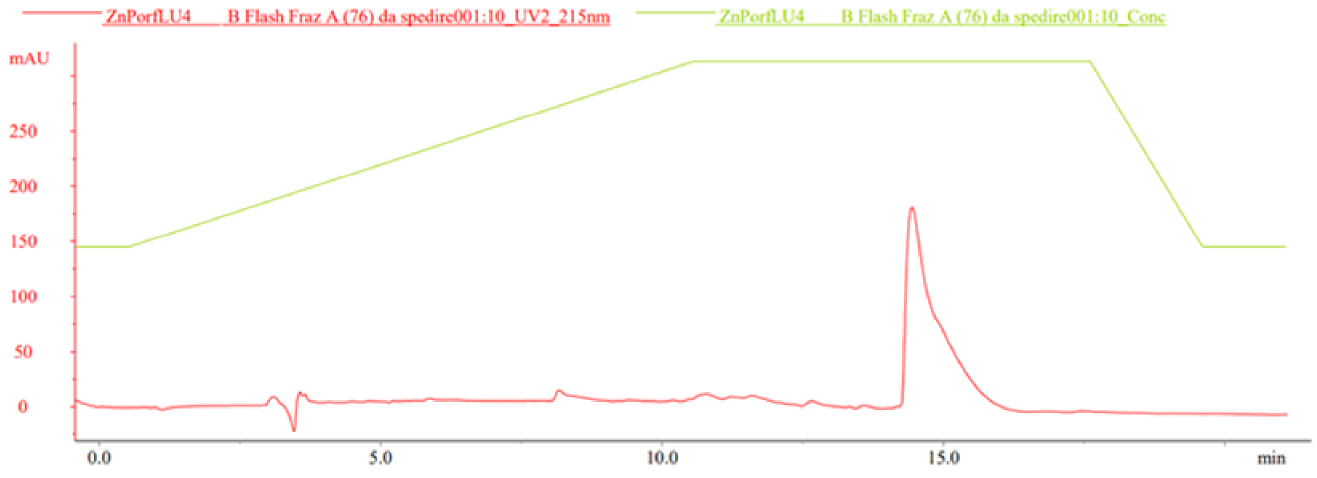

Figure S1. HPLC chromatogram of [**4**]. Column: Jupiter 5 µ C4 300Å – Gradient: 50-100 %B in 10 min – Flow: 1mL/min - Detection UV: 215 nm - R_t: 14.4 min; Purity=89%

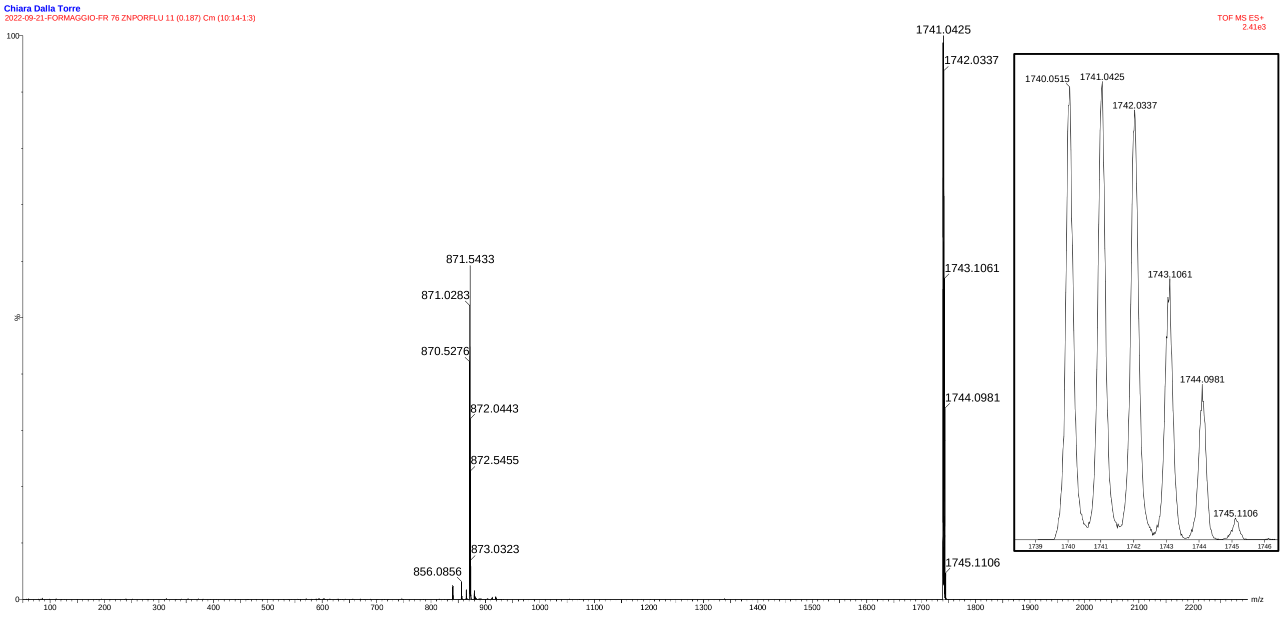

Figure S2. ESI-HRMS spectrum of [**4**]. Calculated m/z values: $[M+H]^+$=1739.877; $[M+2H]^{2+}$=870.4385. A zoom of the molecular peak is reported in the inset.

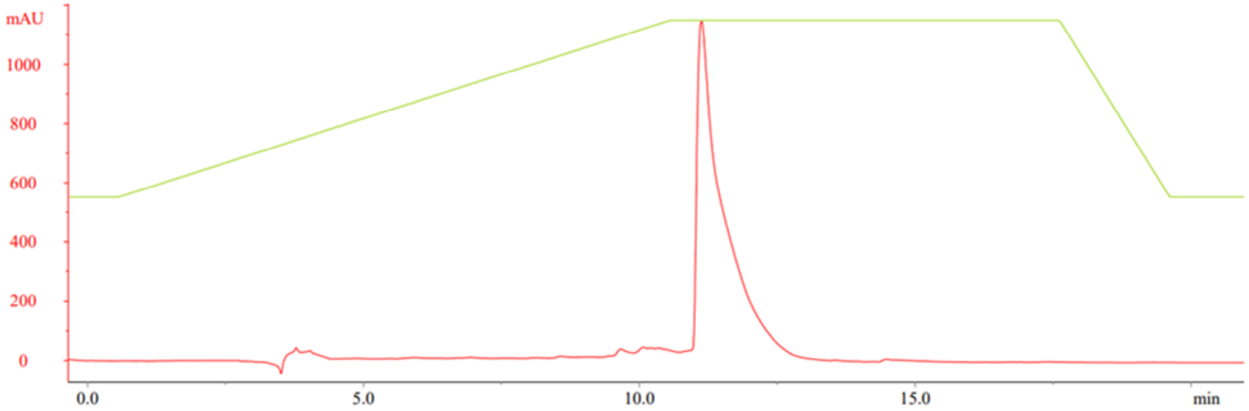

Figure S3. HPLC chromatogram of [**5**]. Column: Jupiter 5 μ C4 300Å – Gradient: 50-100 %B in 7 min – Flow: 1mL/min -R_t = 11.13 min, Purity = 95%

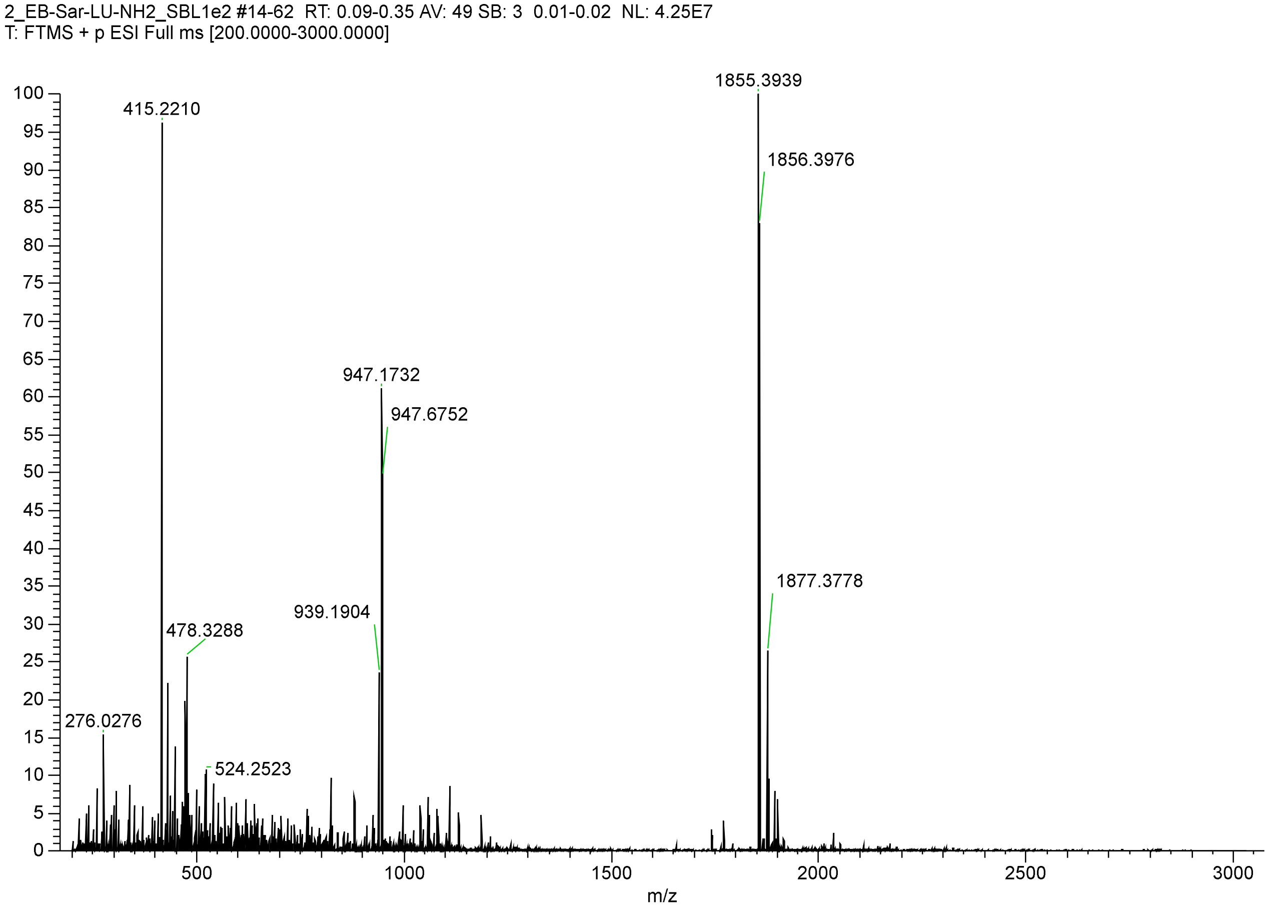

Figure S4. ESI-HRMS spectrum of [**5**]. Calculated m/z values: $[M+H]^+$= 1855.3828; $[M+H+Na]^{2+}$= 939.1914; $[M+H+K]^{2+}$= 947.1914.

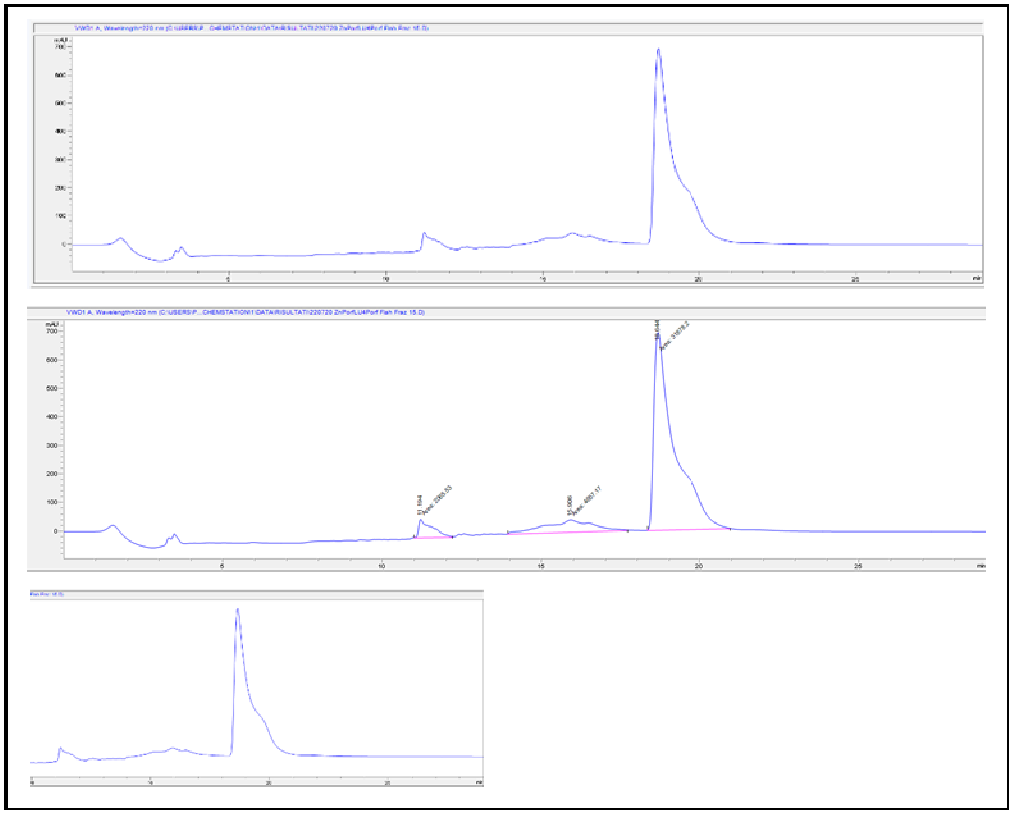

Figure S5. HPLC chromatogram of [**1**]. Column: Jupiter 5 μ C4 300Å – Gradient: 20-100 %B in 28 min – Flow: 1mL/min - Detection UV: 220 nm - Rt: 18.64 min; Purity=81%

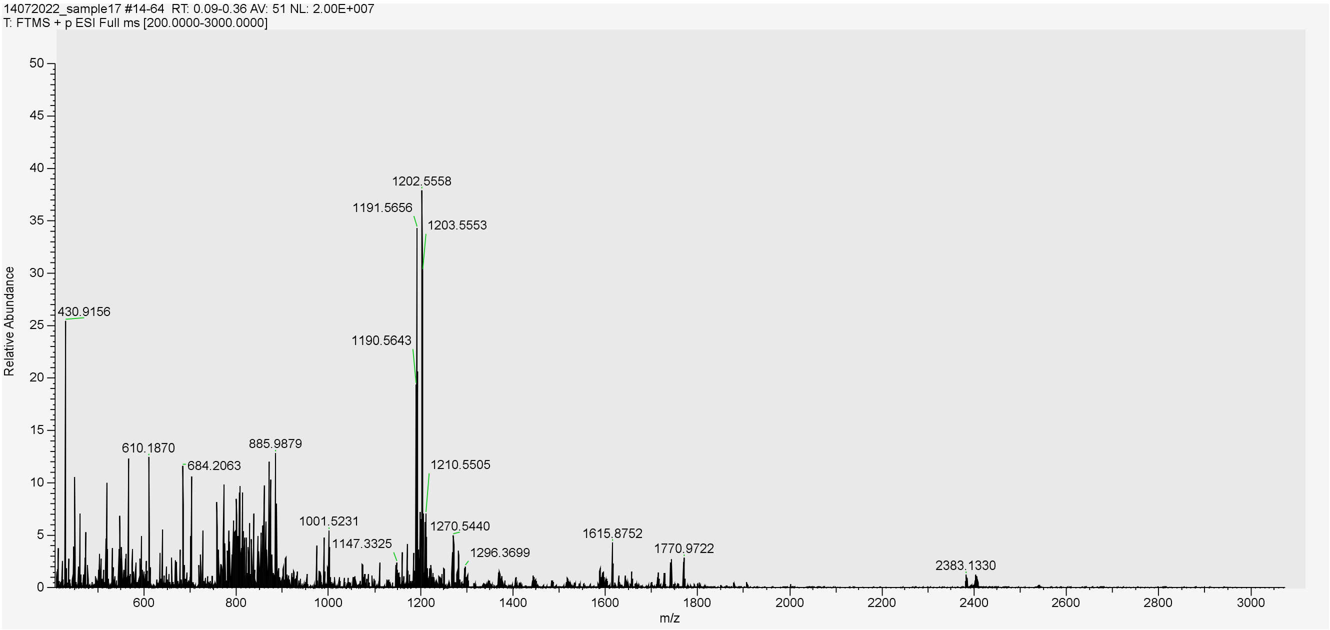
14072022_sample17 #14-64 RT: 0.09-0.36 AV: 51 NL: 2.00E+007
T: FTMS + p ESI Full ms [200.0000-3000.0000]
Relative Abundance
m/z
1202.5558
1191.5656
1203.5553
430.9156
1190.5643
610.1870
885.9879
684.2063
1210.5505
1001.5231
1270.5440
1147.3325
1296.3699
1615.8752
1770.9722
2383.1330

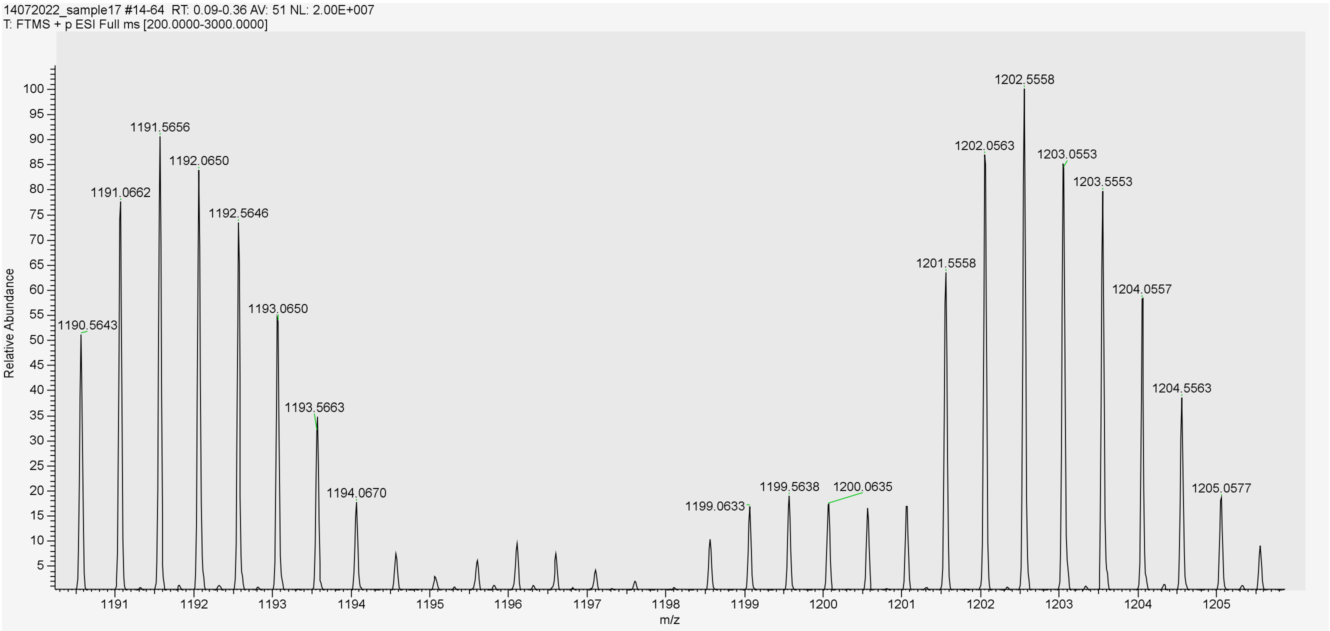
14072022_sample17 #14-64 RT: 0.09-0.36 AV: 51 NL: 2.00E+007
T: FTMS + p ESI Full ms [200.0000-3000.0000]
Relative Abundance
m/z
1202.5558
1191.5656
1202.0563
1203.0553
1192.0650
1203.5553
1191.0662
1192.5646
1201.5558
1204.0557
1193.0650
1190.5643
1204.5563
1193.5663
1194.0670
1199.5638
1200.0635
1205.0577
1199.0633

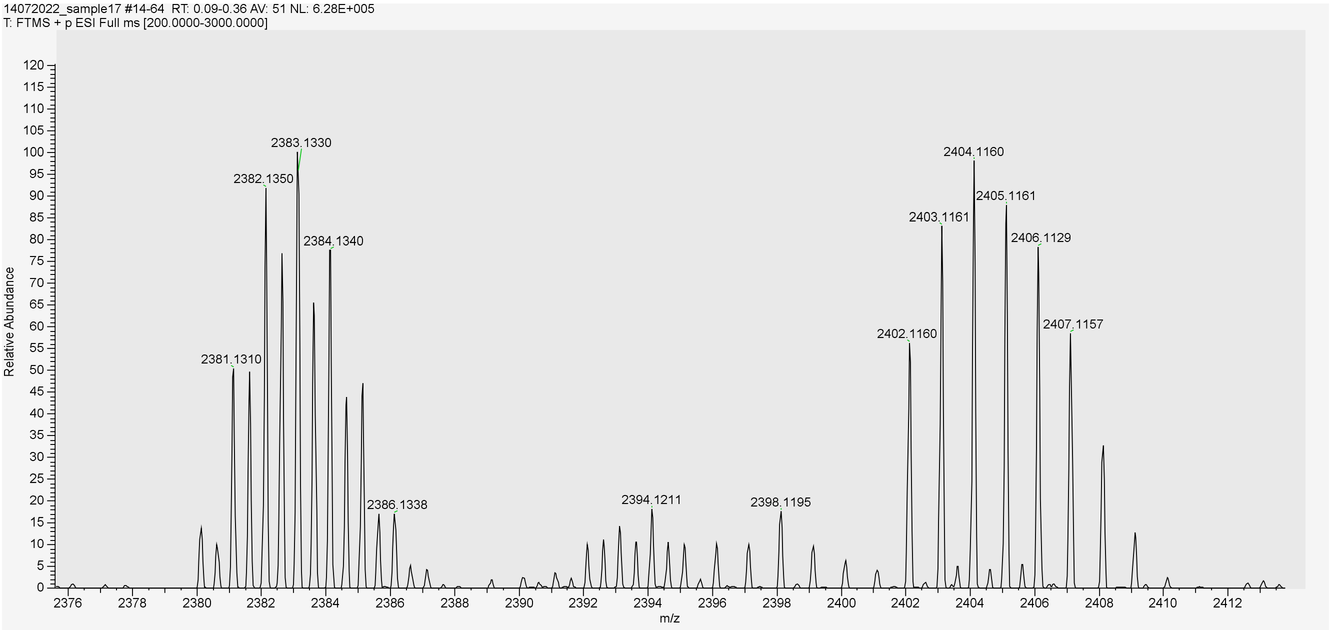

Figure S6. ESI-HRMS spectrum (with selected portions) of [**1**]. Calculated m/z values: $[M+H]^{+}$=2380.1134; $[M+Na]^{+}$=2402.1034; $[M+2H]^{2+}$=1190.5567; $[M+H+Na]^{2+}$=1201.5517.

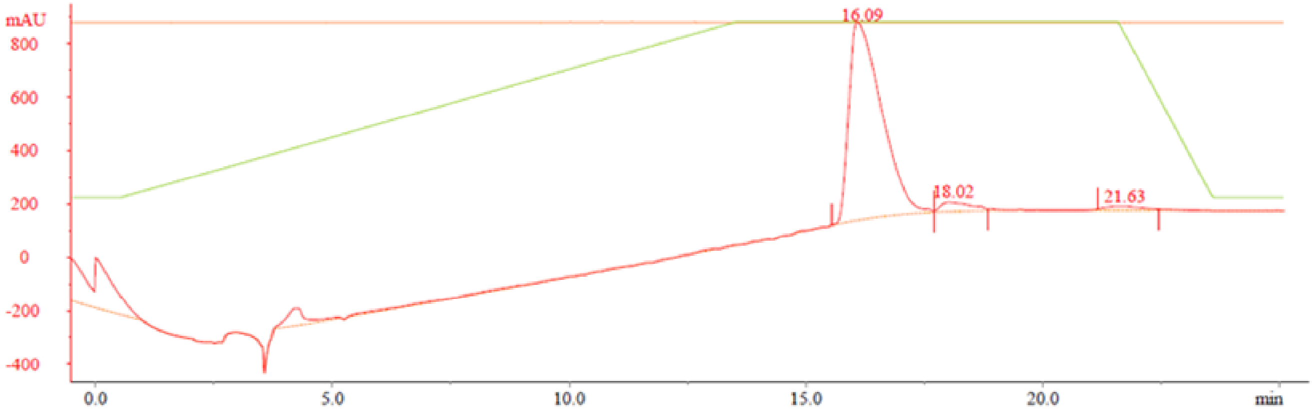

No	Ret min	Area mAU*min	Area/Peak area (time) %
1	16.09	590.4519	93.50
2	18.02	26.2801	4.16
3	21.63	14.7447	2.33

Figure S7. HPLC chromatogram of [**2**]. Column: Jupiter 5 µ C4 300Å – Gradient: 50-100 %B in 26 min – Flow: 1mL/min -R_t = 16.09 min, Purity = 93.5%

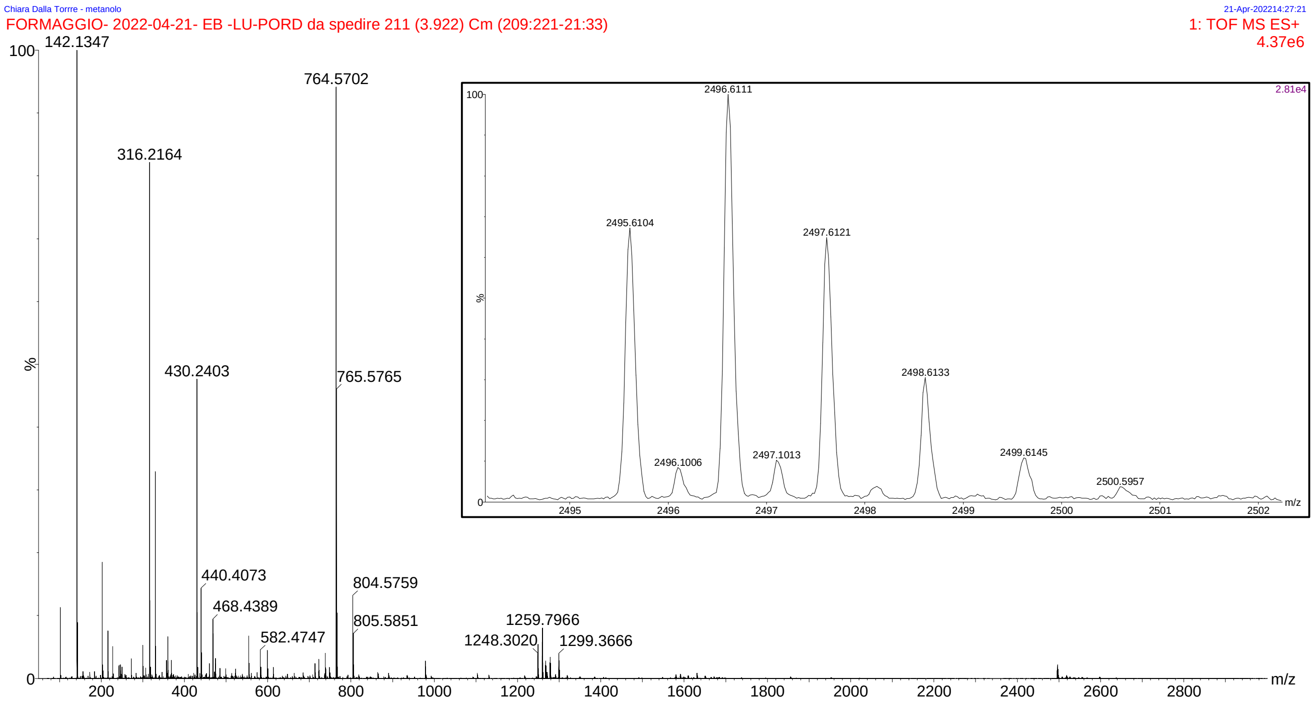

Figure S8. ESI-HRMS spectrum of [**2**]. Calculated m/z values: $[M+H]^{+}$= 2495.6091; $[M+2H]^{2+}$= 1248.3045; $[M+H+Na]^{2+}$= 1259.4595. A zoom of the molecular peak is reported in the inset.

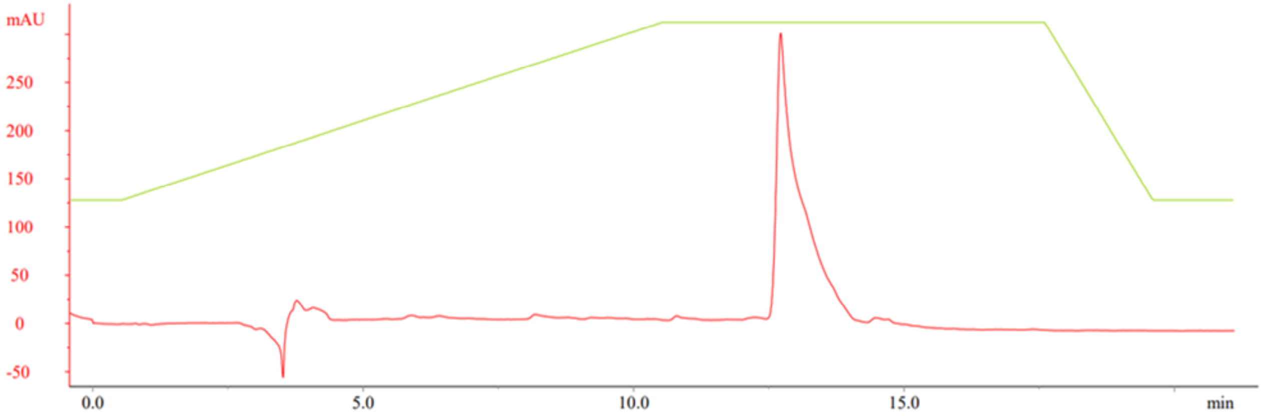

Figure S9. HPLC chromatogram of [**3**]. Column: Jupiter 5 µ C4 300Å – Gradient: 50-100 % B in 7 minutes (green) – Flow: 1mL/min -Rf = 12.72 min, Purity = 95%

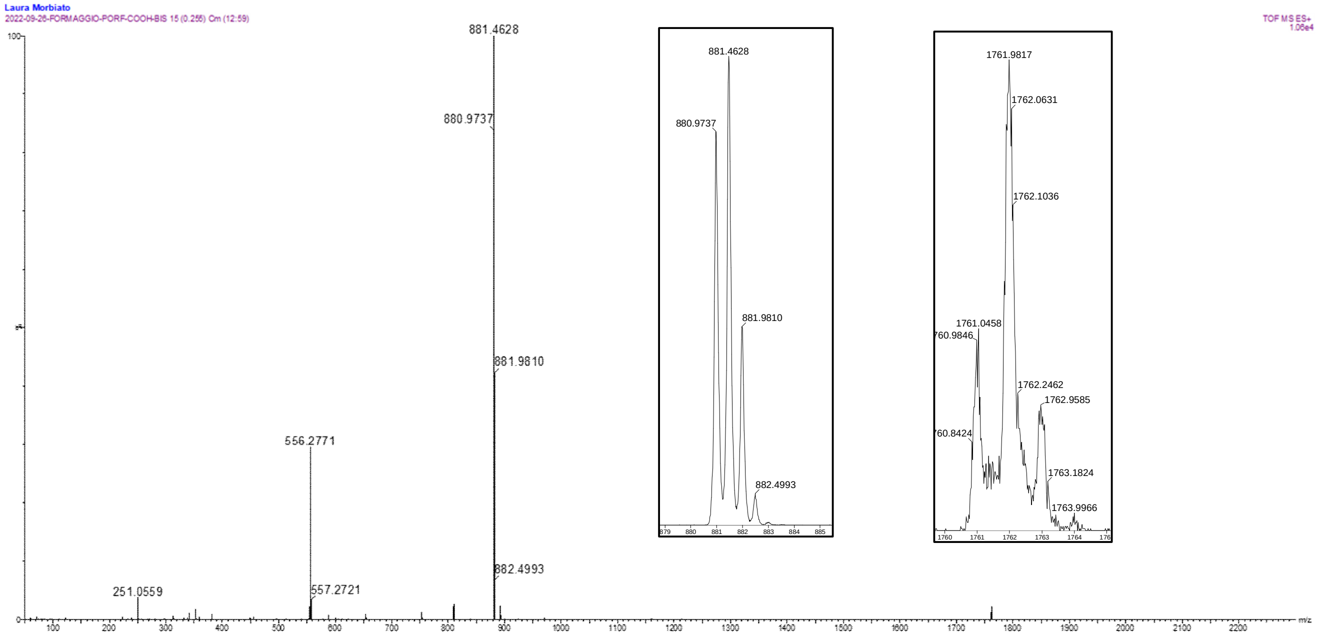

Figure S10. ESI-HRMS spectrum of [**3**]. Calculated m/z values: $[M+H]^{+}$=1760.99; $[M+2H]^{2+}$=880.99. A zoom of selected portions is reported in the insets.

S2.2. Characterization of the free chromophores

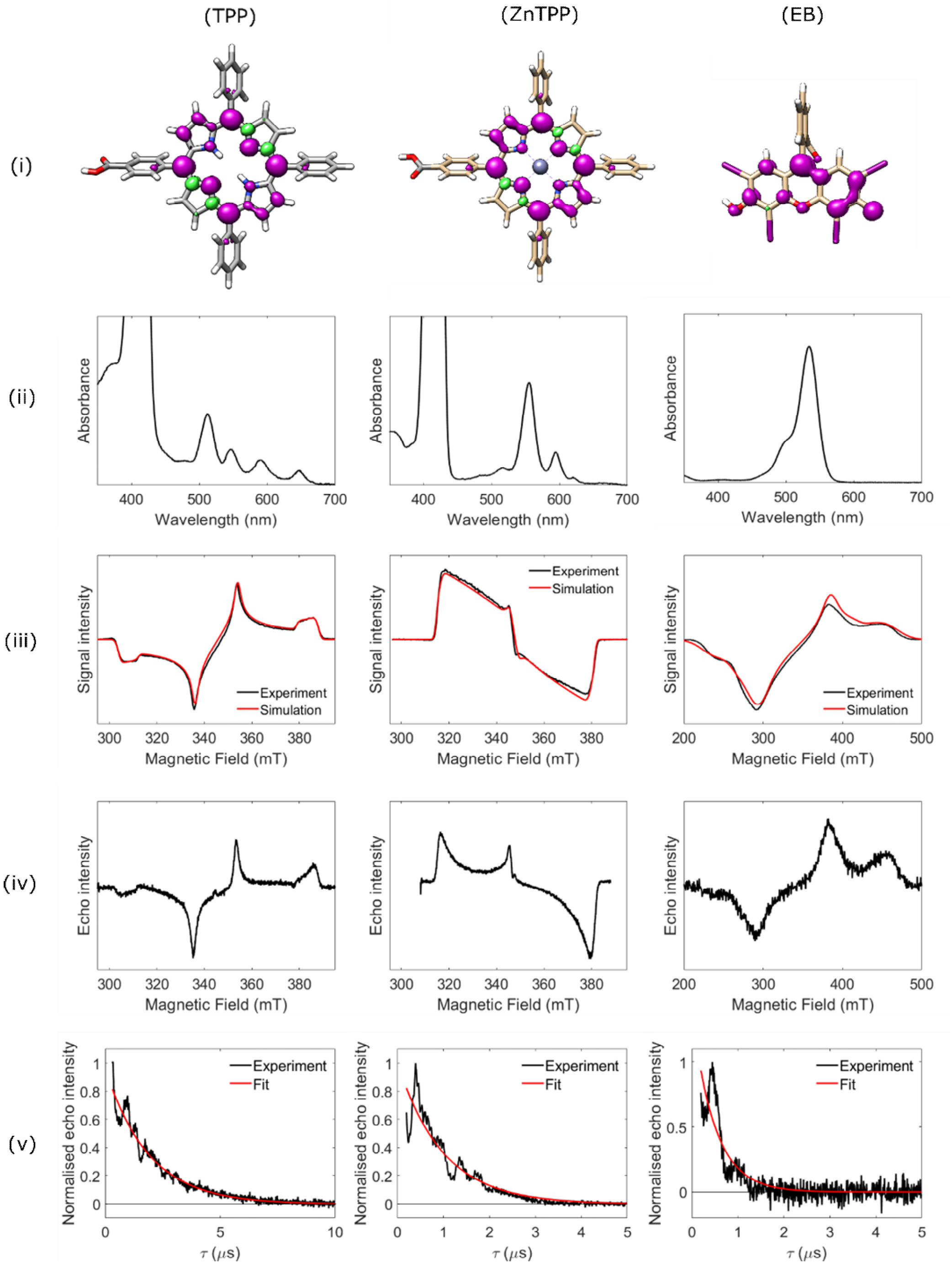

Figure S11. Characterization of the free chromophores: TPP, ZnTPP and EB. (i) Triplet state electronic spin density distributions calculated by DFT, functional B3LYP, basis sets Def2SVP (for H, C, N and O) and DefTZVP (for Zn and I). (ii) Room-temperature UV-Vis absorption

spectra in ethanol. (iii) trESR spectra averaged between 0.4 and 0.9 μs after laser excitation (black), and simulations using *EasySpin pepper* function[4] (red) with the parameters from Table S3. (iv) Echo-detected field-swept spectra after laser excitation. (v) Phase-memory time experiments after laser excitation measured on the most intense ESR feature (black) with monoexponential fits (red) yielding T_m values of (1.87±0.03) μs, (0.84±0.04) μs and (0.35±0.04) μs for TPP, ZnTPP and EB, respectively. The laser excitation wavelengths were 512 nm, 556 nm and 532 nm for TPP, ZnTPP and EB, respectively. The observed ESEEM frequencies are (TPP) 2.9 MHz, (ZnTPP) 3.2 MHz and (EB) 1.8 MHz, and the field positions at which the phase-memory time experiments were recorded are: (TPP) 335.4 mT, (ZnTPP) 380.0 mT and (EB) 290.0 mT. it can therefore be concluded that the main nuclei contributing to the ESEEM oscillations are deuterium from the solvent in the case of EB and nitrogen within the porphyrin ring in the case of TPP and ZnTPP.

Table S3. Parameters used for the simulation of trESR spectra.

	|D| (MHz)	|E| (MHz)	$p_x : p_y : p_z$
TPP	1183	228	0.30 : 0.53 : 0.17
ZnTPP	933	278	0.29 : 0.30 : 0.41
EB	3486	328	0.40 : 0.60 : 0.00

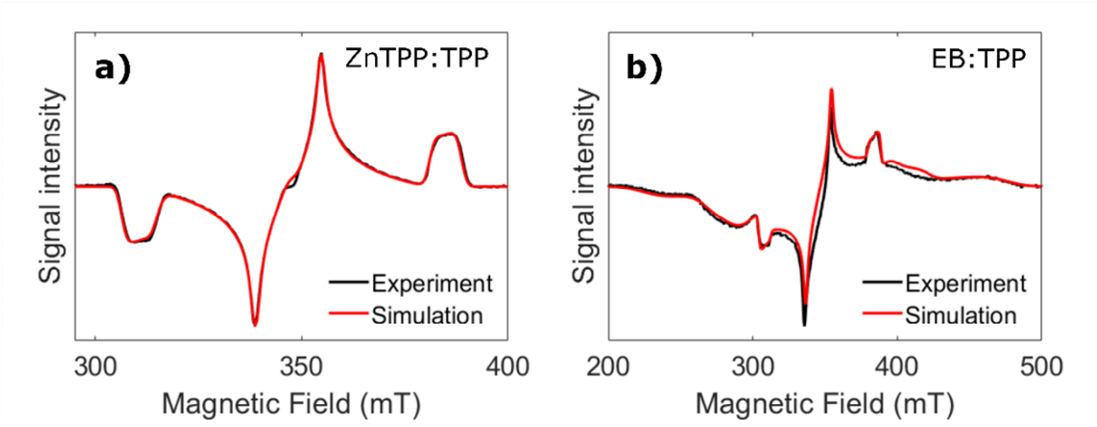

Figure S12. trESR spectra of 1:1 mixtures of ZnTPP:TPP (a) and EB:TPP (b) after laser excitation at 512 nm (black), and simulations using mixtures of the individual triplet spectra in Fig. S11 (iii), performed with *EasySpin pepper* function[4] (red). The fraction of TPP triplet included in the simulations was 65 % in both cases.

S2.3. Characterization of [1] and [2]

No appreciable changes in the optical absorption spectra of the chromophores was observed upon peptide labelling with respect to the free chromophores.

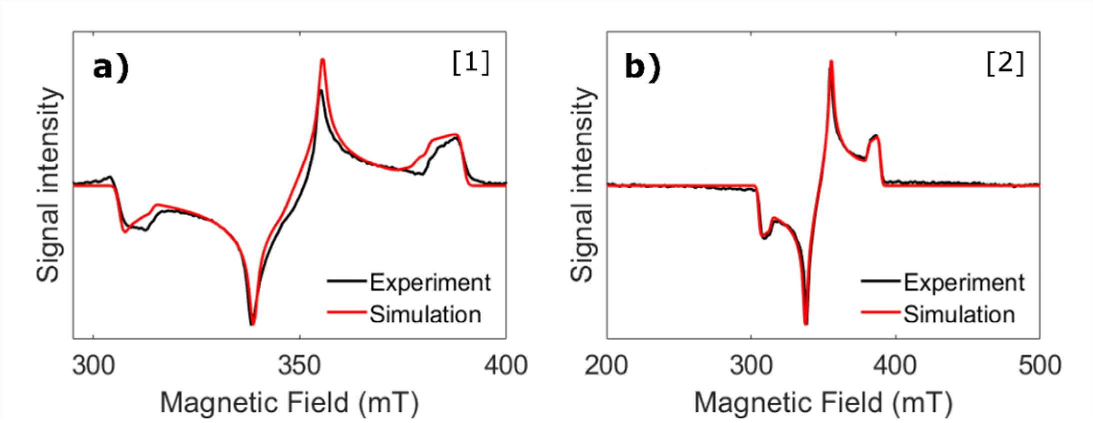

Figure S13. trESR spectra of [**1**] (a) and [**2**] (b) after laser excitation at 512 nm (black), and simulations using mixtures of the individual triplet spectra in Fig. S11 (iii), performed with *EasySpin pepper* function[4] (red). The fractions of TPP triplet included in the simulations were 85 % (a) and 100 % (b).

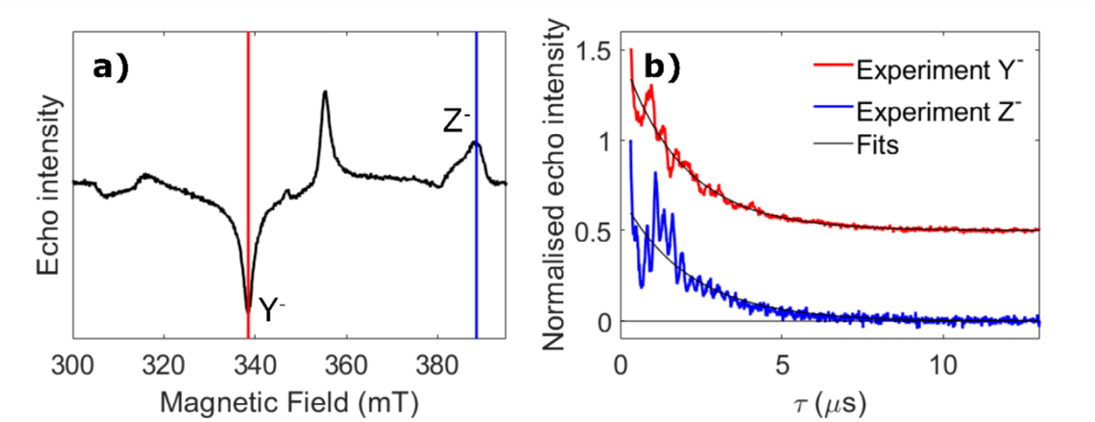

Figure S14. Pulsed ESR characterization of [**1**] after laser excitation at 512 nm. (a) Echo-detected field-swept spectrum indicating the field positions used for the spin relaxation experiments. (b) Phase-memory time experiments (color) with monoexponential fits (black) yielding T_m values of (1.91 ± 0.04) μs and (2.1 ± 0.1) μs. Peptide [**2**] yielded the same T_m values as peptide [**1**].

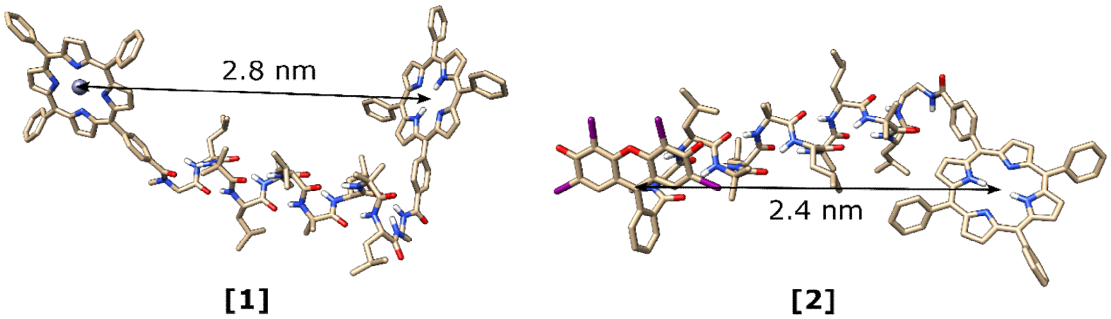

Figure S15. DFT-optimized geometries of [**1**] and [**2**], with the chromophore center-to-center distances indicated. Calculations were carried out with the functional PBE1PBE and the basis set 6-31g(d), replacing ZnTPP by TPP in [**1**] and I by H in [**2**].

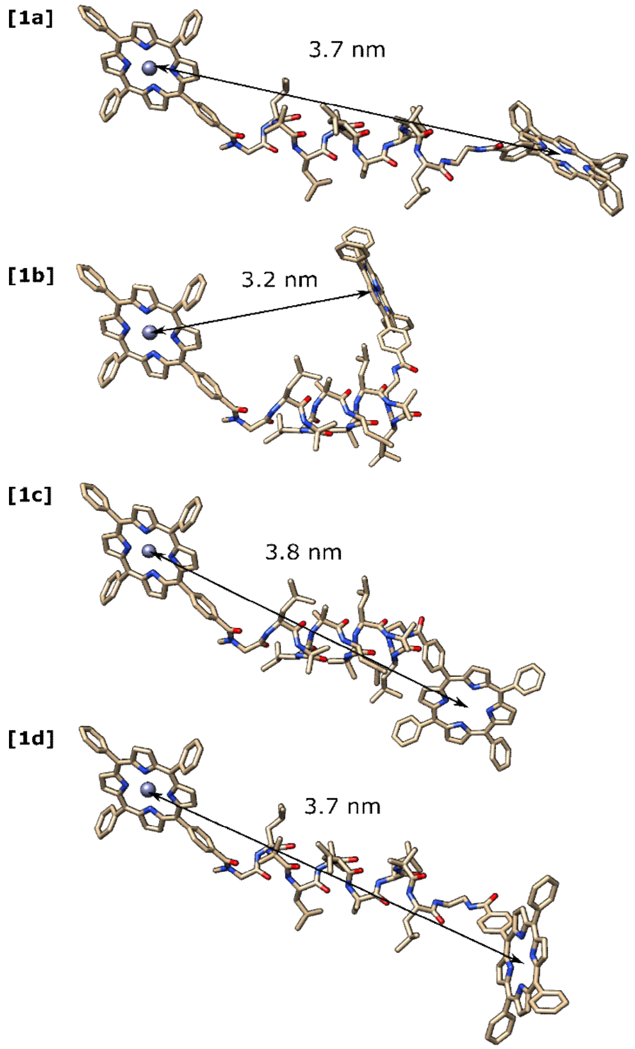

Figure S16. Higher-energy DFT-optimized geometries of [**1**], with the chromophore center-to-center distances indicated. Calculations were carried out with the functional PBE1PBE and the basis set 6-31g(d), replacing ZnTPP by TPP.

[2a]

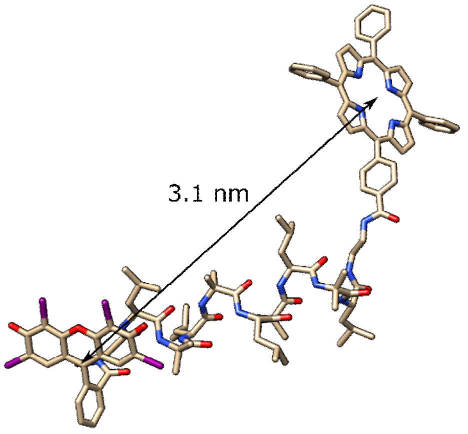

[2b]

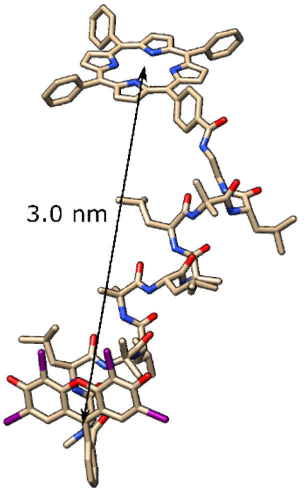

[2c]

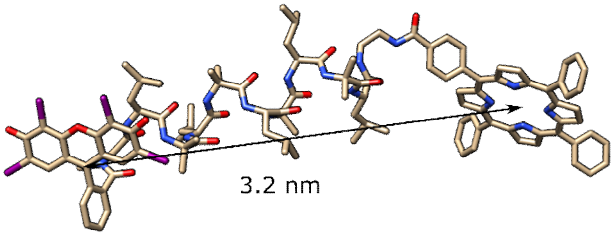

Figure S17. Higher-energy DFT-optimized geometries of [**2**], with the chromophore center-to-center distances indicated. Calculations were carried out with the functional PBE1PBE and the basis set 6-31g(d), replacing I by H.

Table S4. Energies and chromophore–chromophore distances for the structures shown in Fig. S16 and Fig. S17. The energies are relative to the most stable geometry of each molecule (shown in Fig. S15). It should be noted that some of these structures are within or close to the thermal energy of the system 2.48 kJ/mol at 298 K from the minimum energy structure found, and therefore may be thermally populated.

Structure	Energy (kJ/mol)	Distance (nm)
[1a]	31.7	3.7
[1b]	38.3	3.2
[1c]	7.4	3.8
[1d]	0.6	3.7
[2a]	30.9	3.1
[2b]	17.2	3.0
[2c]	2.7	3.2

S2.4. LITTER analysis

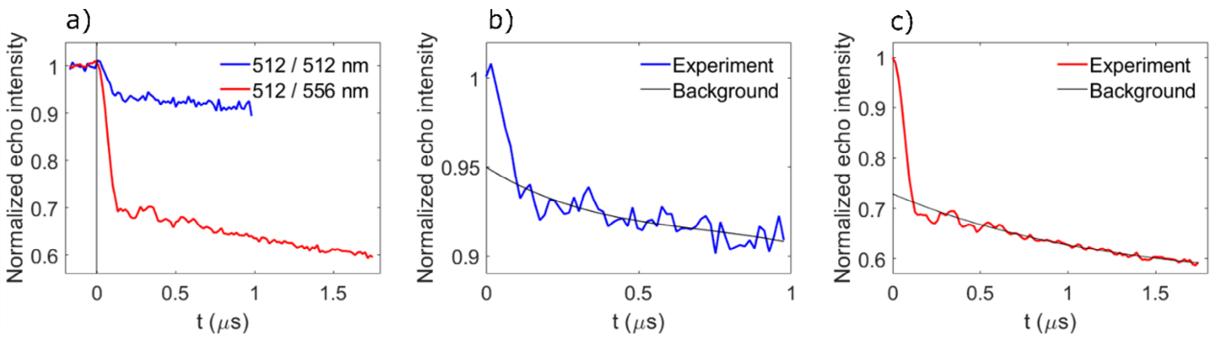

Figure S18. Analysis for the LITTER traces shown in Fig. 2b. (a) Raw data. (b) Third-order polynomial background correction for the 512 / 512 nm trace. (c) Third-order polynomial background correction for the 512 / 556 nm trace. The backgrounds were fitted in DEERAnalysis2022.[5]

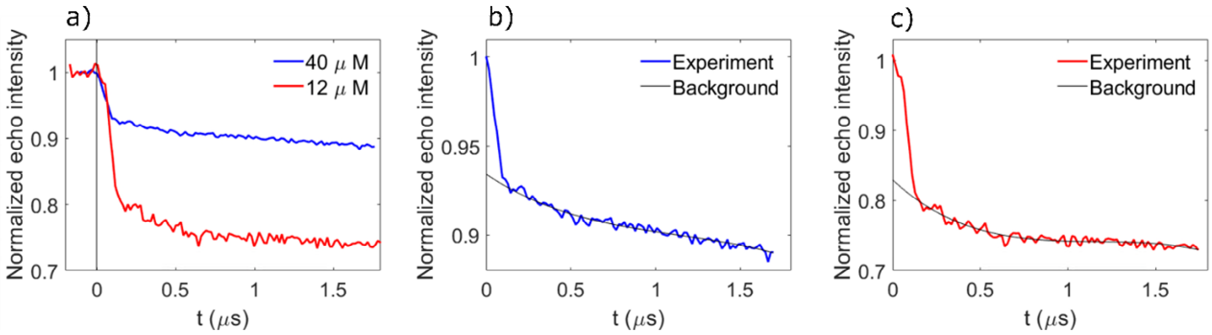

Figure S19. Analysis for the LITTER traces shown in Fig. 3b. (a) Raw data. (b) Third-order polynomial background correction for the 40 μM trace. (c) Third-order polynomial background correction for the 12 μM trace. The backgrounds were fitted in DEERAnalysis2022.[5]

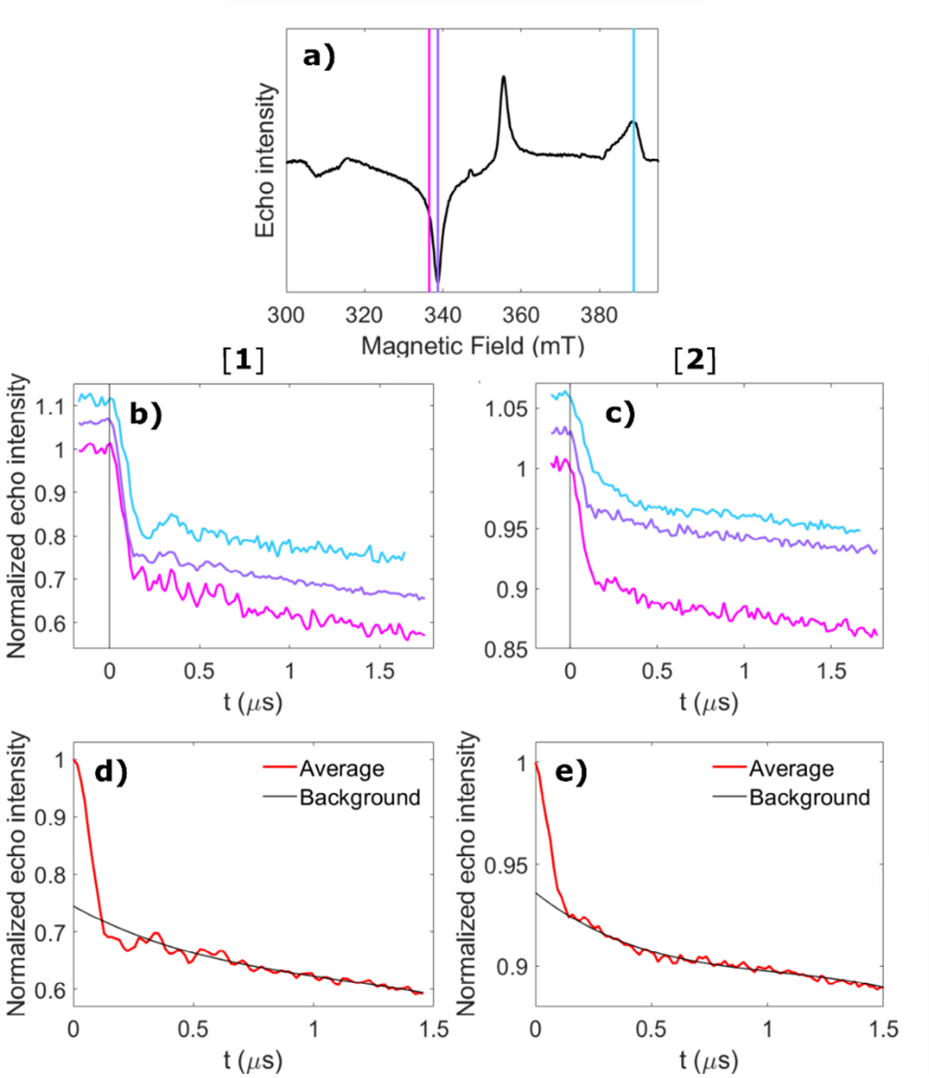

Figure S20. Averaging of 2-color LITTER datasets of 40 μM [**1**] (left) and 40 μM [**2**] (right). (a) Echo-detected field-swept spectrum of [**1**] after laser excitation at 512 nm, indicating the field position used for LITTER with both molecules. (b, c) 2-color LITTER traces of [**1**] (512 / 556 nm) and [**2**] (512 / 532 nm). The middle trace in b is the red trace from Fig. 2b, and the middle trace in c is the blue trace from Fig. 3b. (d, e) Averaged traces (red) and order-3 polynomial backgrounds fitted in DEERAnalysis2022.[5]

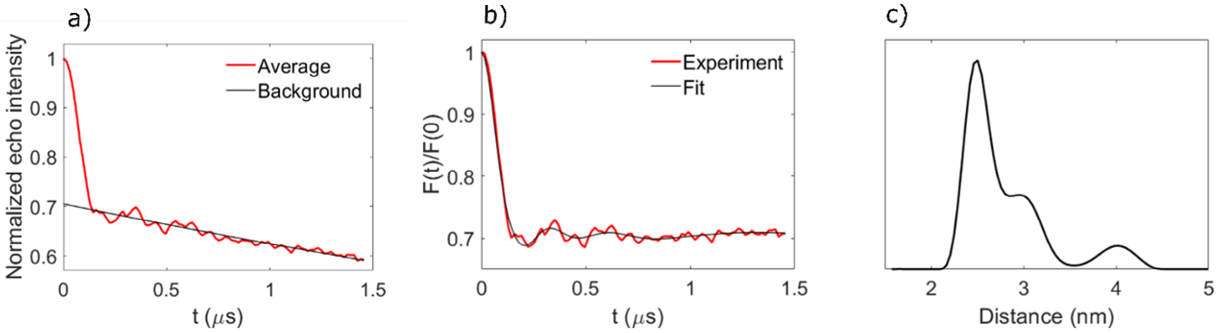

Figure S21. Analysis of the averaged 2-color LITTER trace for 40 μM [**1**] using a three-dimensional homogeneous exponential background. (a) Averaged trace (red) and fitted background (black). (b) Background-corrected trace (red) and fit by Tikhonov regularization, using a regularization parameter α=5. (c) Corresponding distance distribution. The analysis was carried out using DEERAnalysis2022.[5]

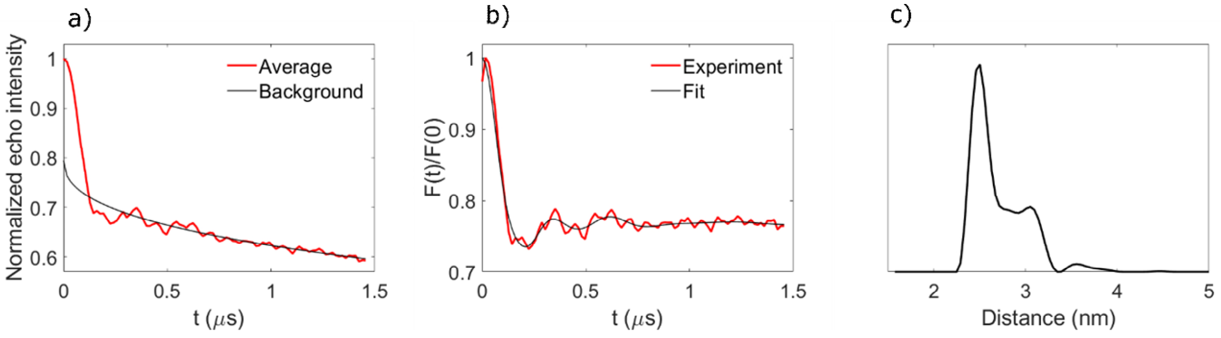

Figure S22. Analysis of the averaged 2-color LITTER trace for 40 μM [**1**] using a stretched exponential background of fitted dimensionality. (a) Averaged trace (red) and background (black) fitted allowing the dimensionality factor to be fitted too. The optimal dimensionality factor was 1.32 (b) Background-corrected trace (red) and fit by Tikhonov regularization, using a regularization parameter α=2. (c) Corresponding distance distribution. The analysis was carried out using DEERAnalysis2022.[5]

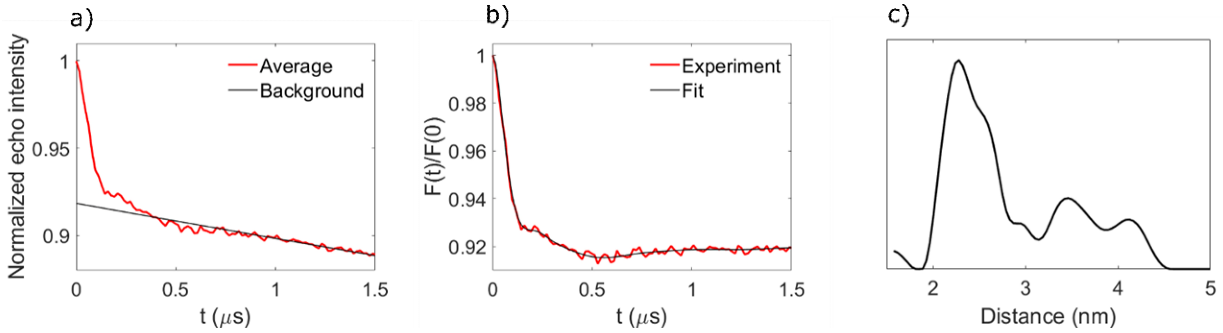

Figure S23. Analysis of the averaged 2-color LITTER trace for 40 μM [**2**] using a three-dimensional homogeneous exponential background. (a) Averaged trace (red) and fitted background (black). (b) Background-corrected trace (red) and fit by Tikhonov regularization, using a regularization parameter α=8. (c) Corresponding distance distribution. The analysis was carried out using DEERAnalysis2022.[5]

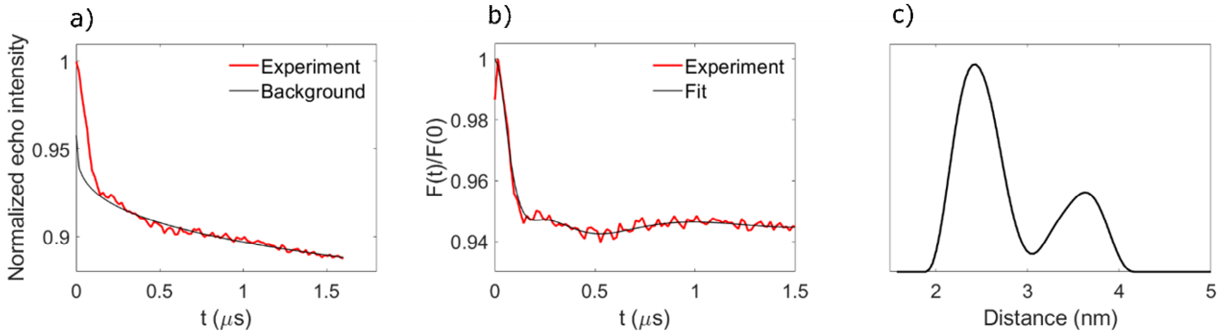

Figure S24. Analysis of the averaged 2-color LITTER trace for 40 μM [**2**] using a stretched exponential background of fitted dimensionality. (a) Averaged trace (red) and background (black) fitted allowing the dimensionality factor to be fitted too. The optimal dimensionality factor was 0.89 (b) Background-corrected trace (red) and fit by Tikhonov regularization, using a regularization parameter α=20. (c) Corresponding distance distribution. The analysis was carried out using DEERAnalysis2022.[5]

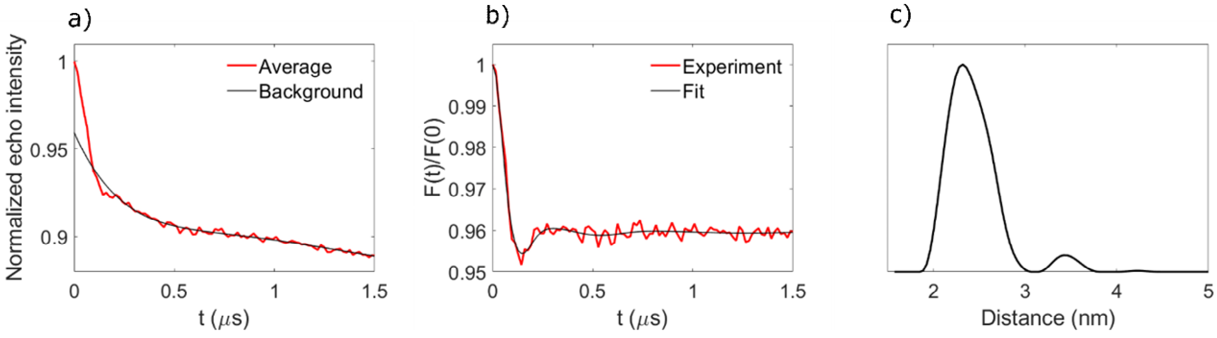

Figure S25. Analysis of the averaged 2-color LITTER trace for 40 μM [**2**] using a fifth-order polynomial background. (a) Averaged trace (red) and fitted background (black). (b) Background-corrected trace (red) and fit by Tikhonov regularization, using a regularization parameter α=10. (c) Corresponding distance distribution. The analysis was carried out using DEERAnalysis2022.[5] Fifth-order polynomial background correction of the 2-color LITTER trace for 40 μM [**1**] failed in DEERAnalysis2022.[5]

Table S5. Modulation depths for the two-color LITTER traces of 40 μM [**1**] and [**2**] obtained with different background corrections in DEERAnalysis2022.[5] Fitting data from system [**1**] with a 5th order polynomial was not possible with uncertainties causing the software to crash.

Background	Modulation depth (%)	
	[**1**]	[**2**]
3D homogeneous	29	8
Stretched exponential with fitted dimensionality	21	4
Order-3 polynomial	26	6
Order-5 polynomial	-	6

S2.5. LITTER modulation depth prediction

Here we derive an analytical expression for the expected modulation depth of LITTER as a function of the excitation efficiencies of each of the chromophores by each of the lasers and the triplet quantum yields of each chromophore. The modulation depth of a PDS experiment is defined as:

$$\Delta = \frac{S_M}{S_0}$$

where S_M is the modulated part of the electron spin-echo intensity and S_0 is the total echo intensity. This expression describes both 1-color and 2-color versions of the experiment.

Considering a general 2-chromophore system A–B, where A and B identify the chromophores whose photogenerated triplet states are used for detection and pumping, we designate the following quantities:

- Excitation efficiency of chromophore α by laser i: e_i^{α}; $\alpha = A, B$; $i = 1{,}2$.
- Triplet QY of chromophore α: Φ_{α}; $\alpha = A, B$.

Both e_i^{α} and Φ_{α} can vary only between 0 and 1.

|In the case of an experiment where A and B are optically identical, either chromophore can be used for detection or pumping and $e_i^A = e_i^B = e_i$ and $\Phi_A = \Phi_B = \Phi_T$

In this case, assuming that all molecules have two chromophores that can be excited, S_0 is calculated as:

$$S_0 = P(T_1^A - S_1^B) + P(S_1^A - T_1^B) + 2P(T_1^A - T_1^B)$$
$$= e_1^A\Phi_A(1 - e_1^B\Phi_B) + e_1^B\Phi_B(1 - e_1^A\Phi_A) + 2e_1^A\Phi_A e_1^B\Phi_B$$
$$= 2(e_1\ \Phi_T(1 - e_1\ \Phi_T)) + 2(e_1\ \Phi_T)^2$$

Where $P(T_1^A - S_1^B)$ is the probability of forming the triplet state on chromophore A while chromophore B remains in the singlet state after the first laser and similarly for the other states.

S_M is the probability that laser 2 induces triplet formation in the $|T_+\rangle$ or $|T_-\rangle$ states for the chromophore in a singlet state after the first laser has induced a triplet state in the other chromophore, causing a |Δm_s |=1, while $|T_0\rangle$, with a Δm_s=0, only contributes to the unmodulated echo intensity.[12,13] Assuming that the likelihood of population the three triplet states is equal:

$$S_M = P(T_1^A - T_2^B, [+,-]) + P(T_2^A, [+,-] - T_1^B)$$
$$= e_1^A\Phi_A(1 - e_1^B\Phi_B)\frac{2}{3}e_2^B\Phi_B + e_1^B\Phi_B(1 - e_1^A\Phi_A)\frac{2}{3}e_2^A\Phi_A$$
$$= 2(e_1\ \Phi_T(1 - e_1\ \Phi_T)\frac{2}{3}e_2\ \Phi_T)$$

In this case the modulation depth is:

$$\Delta = \frac{S_M}{S_0} = \frac{2(e_1\ \Phi_T(1 - e_1\ \Phi_T)\frac{2}{3}e_2\ \Phi_T)}{2(e_1\ \Phi_T(1 - e_1\ \Phi_T)) + 2(e_1\ \Phi_T)^2} = (1 - e_1\ \Phi_T)\frac{2}{3}e_2\ \Phi_T$$

Applying the limits to e_i^α and Φ_α and noting that in this case the same chromophore must be used for both detection and pumping and therefore a balance in the values of e_1 and e_2 is required for optimal signal to noise, assuming that $e_1 = e_2 = 0.5$ the maximum value of the modulation depth is ca. 17%.

To calculate S_0 and S_M in the case of two optically orthogonal chromophores A and B in a 2-colour LITTER experiment where A is used for detection and B is used for pumping, we make the following assumptions:

1. The detection MW pulses are resonant with a part of the triplet ESR spectrum of A where triplet B has negligible intensity and therefore the detected echo is only due to triplets of chromophore A.

2. The excitation efficiencies do not depend on the number of chromophores left in the singlet ground state and are therefore constant throughout the experiment.

3. All molecules have two chromophores that can be photoexcited.

As before, we account for the fact that only molecules where the pump triplet is formed in states $|T_+\rangle$ or $|T_-\rangle$, causing a $|\Delta m_S| = 1$, contribute to the modulated signal, while $|T_0\rangle$, with a $\Delta m_S = 0$, only contributes to the unmodulated echo intensity.[12,13] For the cases with no preferential molecular orientation and no photoselection, such as the experiments carried out in this work, the population of each triplet sublevel is independent of the chromophore and equal to 1/3.

In the 2-colour experiment the modulated part of the signal in LITTER (S_M) arises from molecules where laser 1 forms the triplet of chromophore A, leaving chromophore B in the ground singlet state, and laser 2 subsequently forms the triplet of chromophore B in states $|T_+\rangle$ or $|T_-\rangle$. This modulated signal can be calculated as the product of the probabilities of these two events:

$$S_M = P(T_1^A - S_1^B)\ P(T_2^B, +/-) = e_1^A \Phi_\mathrm{A}(1 - e_1^B \Phi_B)\ \frac{2}{3} e_2^B \Phi_B$$

The total echo intensity is given by the probability of laser 1 forming the triplet of chromophore A, independently of the spin state of chromophore B:

$$S_0 = P(T_1^A) = e_1^A \Phi_A$$

The modulation depth of the 2-colour LITTER experiment is therefore

$$\Delta = \tfrac{2}{3} e_2^B \Phi_B (1 - e_1^B \Phi_B) \qquad (1)$$

Appling the limits that e_2^B and Φ_B must lie between 0 and 1 it is here noted that if we assume chromophore B does not contribute to the detected signal and therefore Δ is maximized when the e_1^B is minimized. The maximum theoretically achievable modulation depth according to equation (1) is 2/3 (66.7%) and it occurs when $e_1^B = 0$, $e_2^B = 1$ and $\Phi_B = 1$. This calculation assumes equal population of the three triplet states $M_S = +1, 0, -1$.

For the experimental conditions used in this and previous works,[3] it is reasonable to assume $e_2^B = 1$. With this and the triplet QY values reported in the literature for TPP, ZnTPP and EB

(0.88, 0.90 and 0.83, respectively),[14] we can plot the expected modulation depths for each of the pump chromophores as a function of their excitation efficiency by laser 1 (e_1^B) (Fig. S26). With the TPP, ZnTPP and EB pump chromophores studied in this work, all with $\Phi_B < 1$, the maximum achievable modulation depths are 0.59, 0.60 and 0.55, respectively.

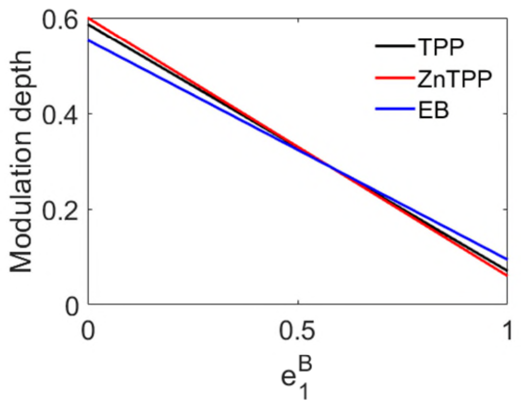

Figure S26. Expected modulation depths for 2-colour LITTER experiments with different pump chromophores: TPP (black), ZnTPP (red) and EB (blue), as a function of the excitation efficiency of the pump chromophore by laser 1 (e_1^B).

The dependence of the modulation depth on Φ_B, for $e_2^B = 1$ and different values of e_1^B, is plotted in Fig. S26. For small values of e_1^B, expected for 2-color experiments with non-identical chromophores, the modulation depth increases with Φ_B. Conversely, for large values of e_1^B, expected for 1-color experiments with identical chromophores, the modulation depth reaches a maximum and decreases as Φ_B approaches 1.

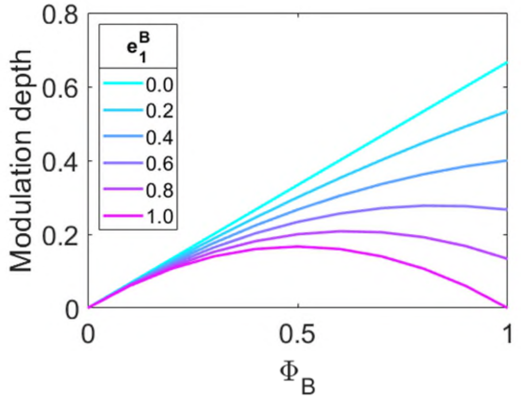

Figure S27. Expected modulation depth for 2-color LITTER as a function of the triplet QY of the pump chromophore (Φ_B), for different values of the excitation efficiency of the pump chromophore by laser 1 (e_1^B).

Using the curves in Fig. S27 and the modulation depth values obtained experimentally for each of the pump chromophores and applying the assumptions given above, we can estimate the corresponding excitation efficiencies e_1^B (Table S6). The e_1^B values obtained are large even for the 2-colour experiments with non-identical chromophores, suggesting that the modulation depth of LITTER can still be increased significantly by further reducing the value of e_1^B through better chromophore choice (i.e. by improving the photoexcitation selectivity of laser 1 towards chromophore A). It should be noted that these calculations assume 100% sample purity and this was observed not to be the case by HPLC (see Section S2 for HPLC results) so the actual values of e_1^B experimentally are likely lower than those calculated below.

Table S6. Estimated excitation efficiencies (e_1^B) for LITTER with different 2-chromophore systems using equation 1 and assuming $e_2^B = 1$. The pump chromophore is indicated in bold. The TPP–TPP system was measured with a 1-color experiment.

System	$\boldsymbol{\Phi_B}$	$\boldsymbol{\Delta}$	$\boldsymbol{e_1^B}$
**TPP**–TPP	0.88[14]	0.10	0.94
**ZnTPP**–TPP	0.90[14]	0.27	0.61
**EB**–TPP	0.83[14]	0.23	0.70

S2.6 Quantum Yield Measurements analysis

The figures below, Fig. S28 and Fig. S29, show the UV-vis absorption spectra and fluorescence emission spectra used in the calculation of the quantum yields of EB-pep system [5] and TPP-pep system [3] following Equation S3. The parameters used in the calculation of the quantum yield are tabulated in Table S7. Details on the experimental set up used are provided in Section S1.5.

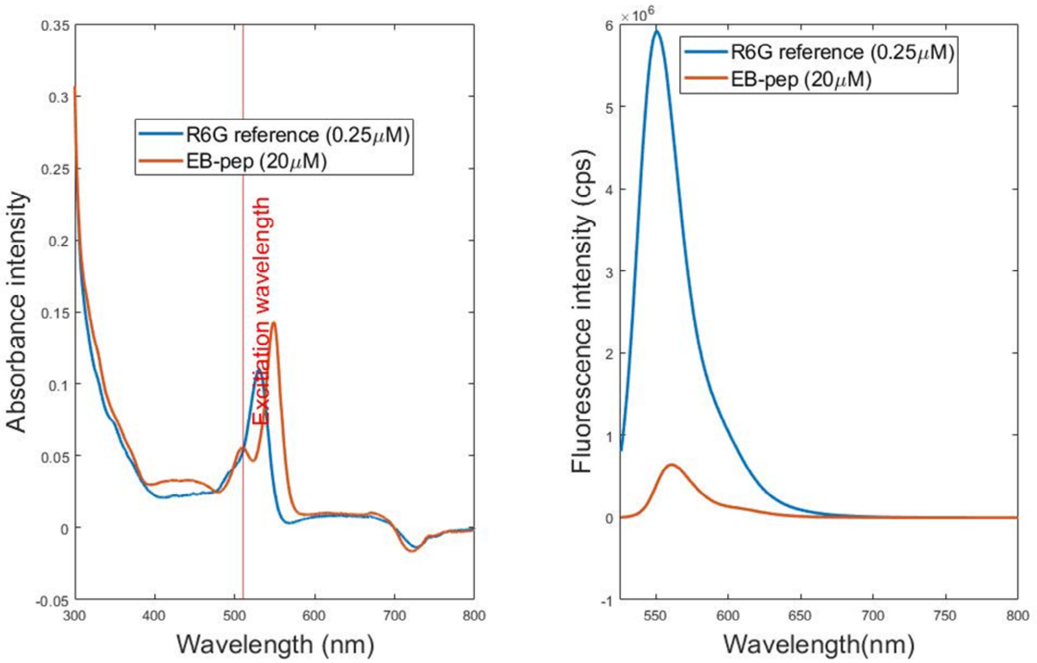

Figure S28. Left: UV-vis absorption spectra for the Rhodamine 6G (R6G, blue line) reference at 0.25 μM and EB-pep system [5] (EB, orange line) at 20 μM. The vertical line marks 510 nm the wavelength used for excitation in the fluorescence measurements. Right: Fluorescence emission spectra for the Rhodamine 6G reference (R6G, blue line) at 0.25 μM and EB-pep system [5] (EB, orange line) at 20 μM. Integrated fluorescence intensity were calculated between 525 nm and 800 nm.

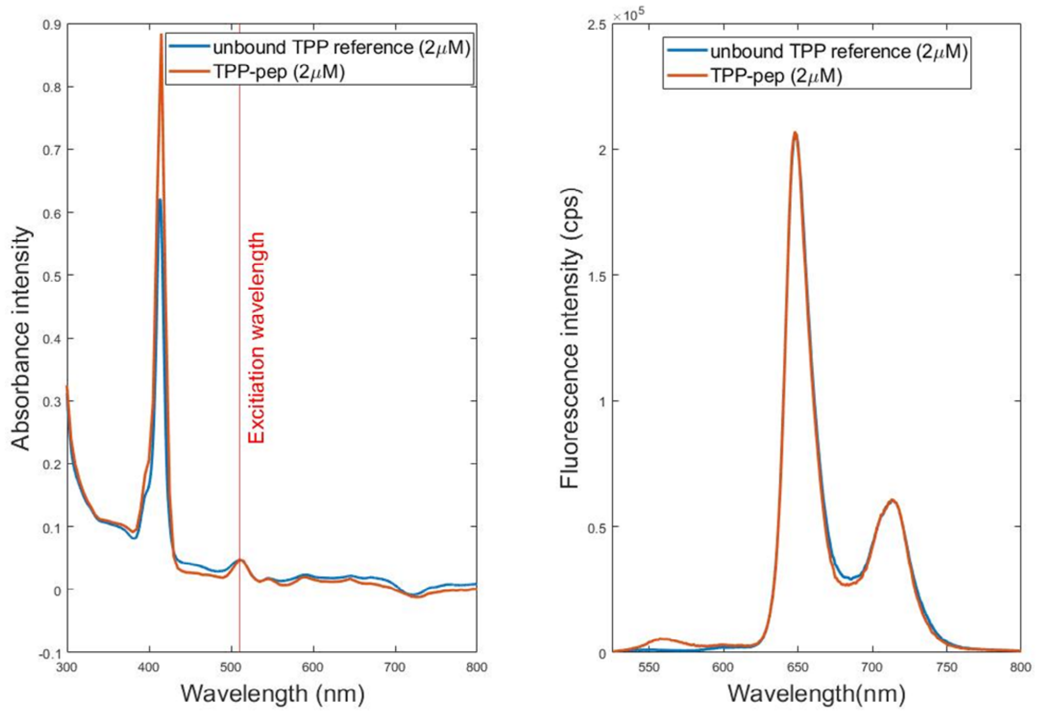

Figure S29. Left: UV-vis absorption spectra for the unbound TPP reference (unbound TPP, blue line) at 2 μM and TPP-pep system [5] (TPP, orange line) at 2 μM. The vertical line marks 510 nm the wavelength used for excitation in the fluorescence measurements. Right: Fluorescence emission spectra for the unbound TPP reference (unbound TPP, blue line) at 2 μM and TPP-pep system [5] (TPP, orange line) at 2 μM. Integrated fluorescence intensity were calculated between 525 nm and 800 nm.

Table S7. Parameters used in the fluorescence quantum yield for EB-pep [**5**] and TPP-pep [**3**] relative to the references Rhodamine 6G and unbound TPP respectively.

System	Reference	$\Phi_{fl,R}$	I	I_R	OD (a.u.)	OD_R (a.u.)	n	n_R	Φ_{fl}
EB-pep [**5**] (20 μM)	Rhodamine 6G (0.25 μM)	0.95	2.5 x 10^6	2.6 x 10^7	0.055	0.053	1.35	1.35	0.095
TPP-pep [**3**] (2 μM)	Unbound TPP (2 μM)	0.13	7.33 x 10^6	7.39 x 10^6	0.0471	0.0473	1.35	1.35	0.128

S2.7 Fluorescence Anisotropy analysis

Fluorescence anisotropy was calculated for all five systems [1]-[5] according to Equation S4. The individual emission spectra are presented in Fig. S30 to Fig. S34, the G factor corrections used are plotted in Fig. S35 and the wavelength dependent fluorescence anisotropy results for all five systems plotted in Fig. S36.

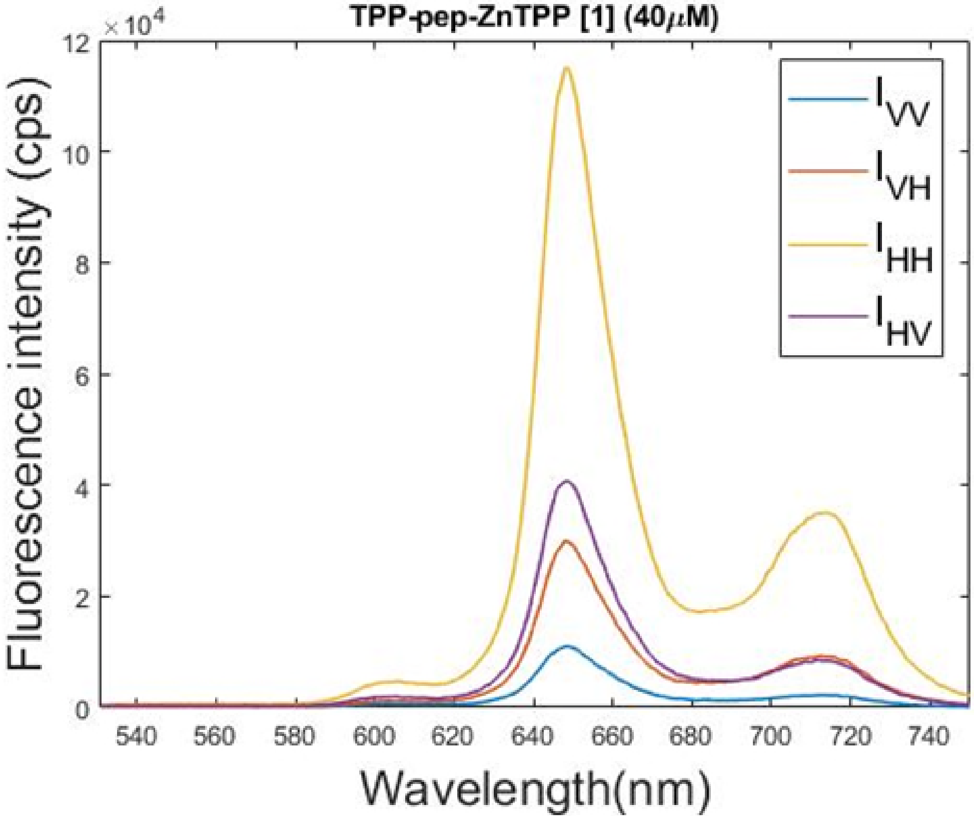

Figure S30. Fluorescence emission spectra for TPP-pep-ZnTPP system [1] (40 μM) recorded with polarizers positioned in the beam path both before (excitation polarizer) and after (emission polarizer) the sample cuvette. Four polarization configurations (V = vertical 0° polarizer rotation, H = horizontal 90° polarizer rotation) were recorded: I_{VV} (λ_{em}), I_{VH} (λ_{em}), I_{HV} (λ_{em}), and I_{HH} (λ_{em}), where the first subscript refers to the polarization of the excitation beam and the second to the polarization of the emission.

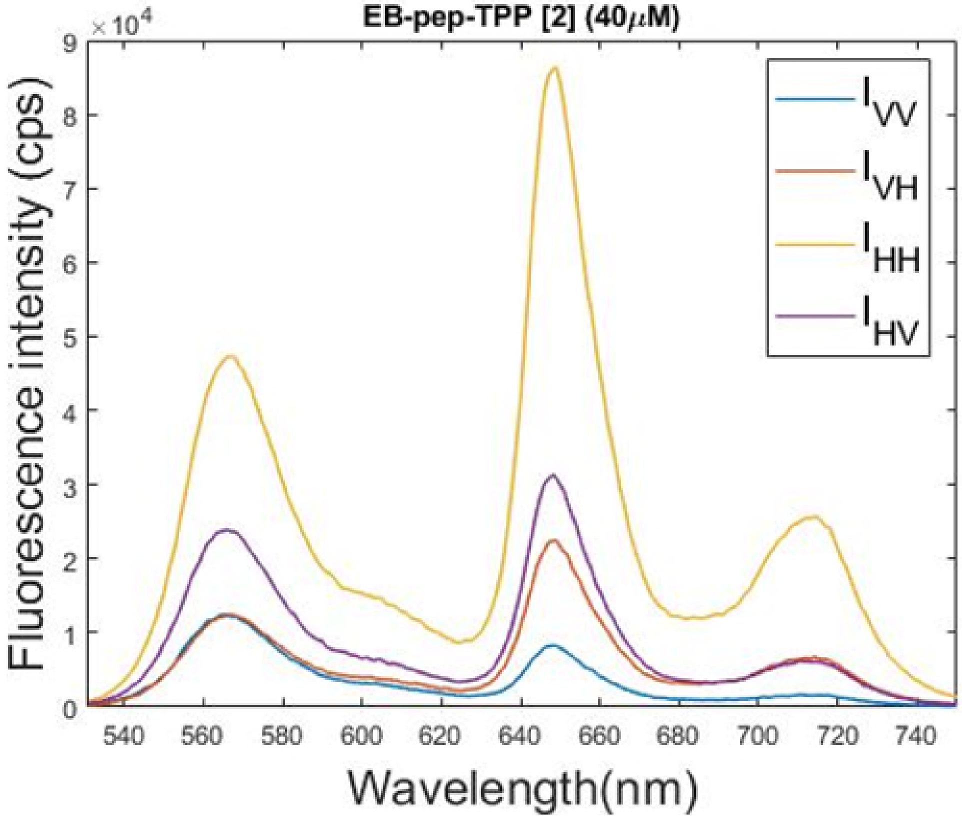

Figure S31. Fluorescence emission spectra for EB-pep-TPP system [**2**] (40 μM) recorded with polarizers positioned in the beam path both before (excitation polarizer) and after (emission polarizer) the sample cuvette. Four polarization configurations (V = vertical 0° polarizer rotation, H = horizontal 90° polarizer rotation) were recorded: I_{VV} (λ_{em}), I_{VH} (λ_{em}), I_{HV} (λ_{em}), and I_{HH} (λ_{em}), where the first subscript refers to the polarization of the excitation beam and the second to the polarization of the emission.

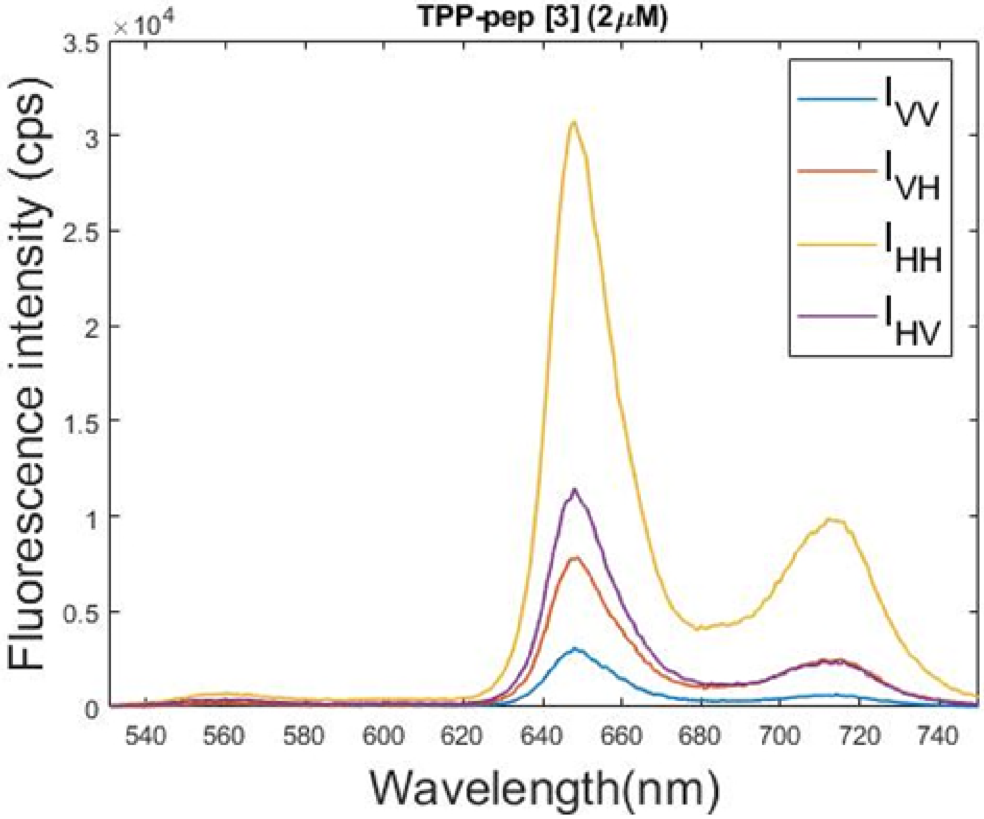

Figure S32. Fluorescence emission spectra for TPP-pep system [**3**] (2 μM) recorded with polarizers positioned in the beam path both before (excitation polarizer) and after (emission polarizer) the sample cuvette. Four polarization configurations (V = vertical 0° polarizer rotation, H = horizontal 90° polarizer rotation) were recorded: I_{VV} (λ_{em}), I_{VH} (λ_{em}), I_{HV} (λ_{em}), and I_{HH} (λ_{em}), where the first subscript refers to the polarization of the excitation beam and the second to the polarization of the emission.

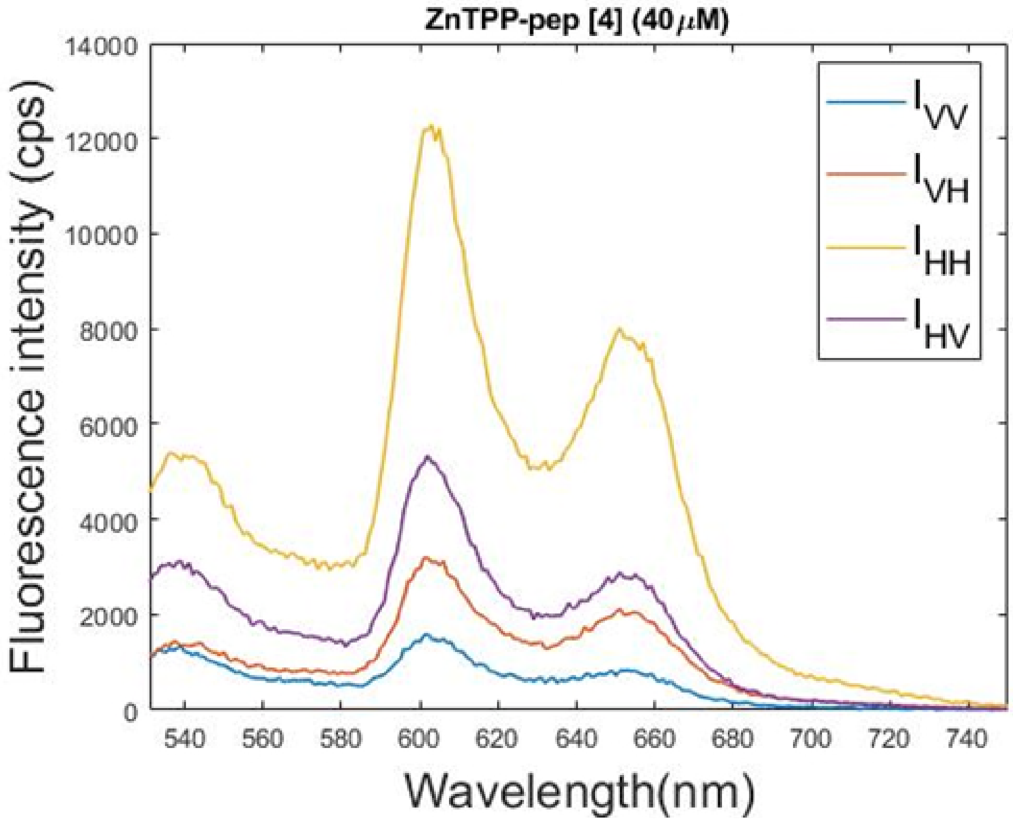

Figure S33. Fluorescence emission spectra for ZnTPP-pep system [**4**] (40 μM) recorded with polarizers positioned in the beam path both before (excitation polarizer) and after (emission polarizer) the sample cuvette. Four polarization configurations (V = vertical 0° polarizer rotation, H = horizontal 90° polarizer rotation) were recorded: I_{VV} (λ_{em}), I_{VH} (λ_{em}), I_{HV} (λ_{em}), and I_{HH} (λ_{em}), where the first subscript refers to the polarization of the excitation beam and the second to the polarization of the emission.

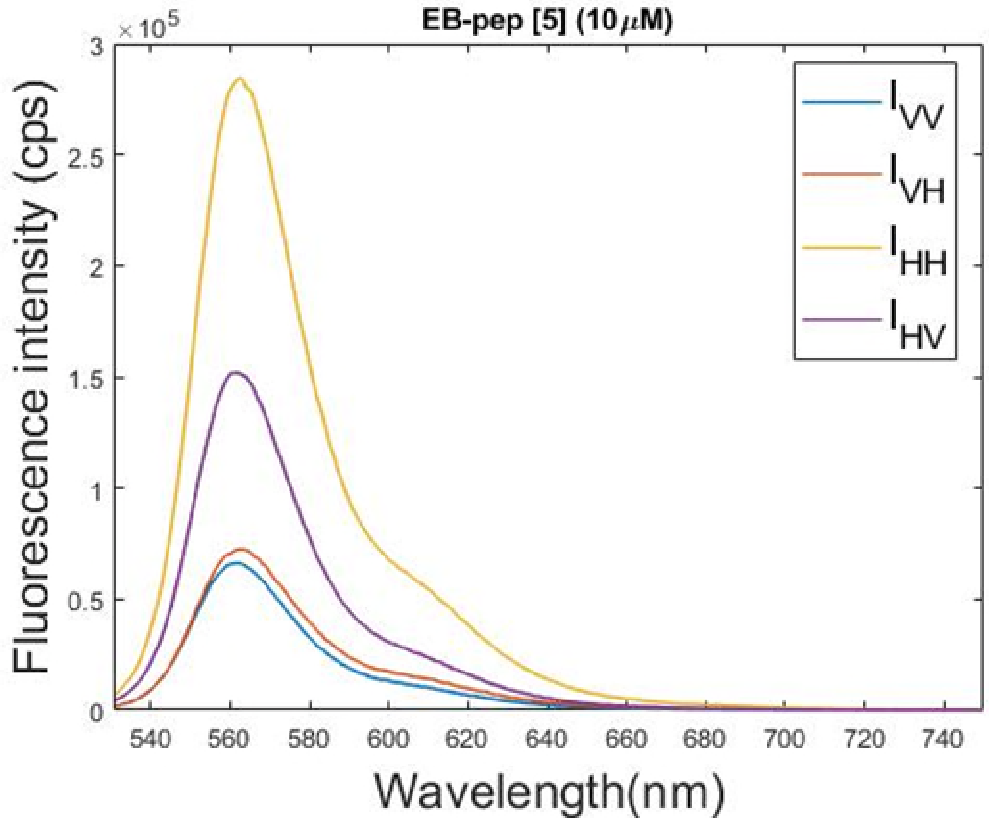

Figure S34. Fluorescence emission spectra for EB-pep system [**5**] (10 μM) recorded with polarizers positioned in the beam path both before (excitation polarizer) and after (emission polarizer) the sample cuvette. Four polarization configurations (V = vertical 0° polarizer rotation, H = horizontal 90° polarizer rotation) were recorded: I_{VV} (λ_{em}), I_{VH} (λ_{em}), I_{HV} (λ_{em}), and I_{HH} (λ_{em}), where the first subscript refers to the polarization of the excitation beam and the second to the polarization of the emission.

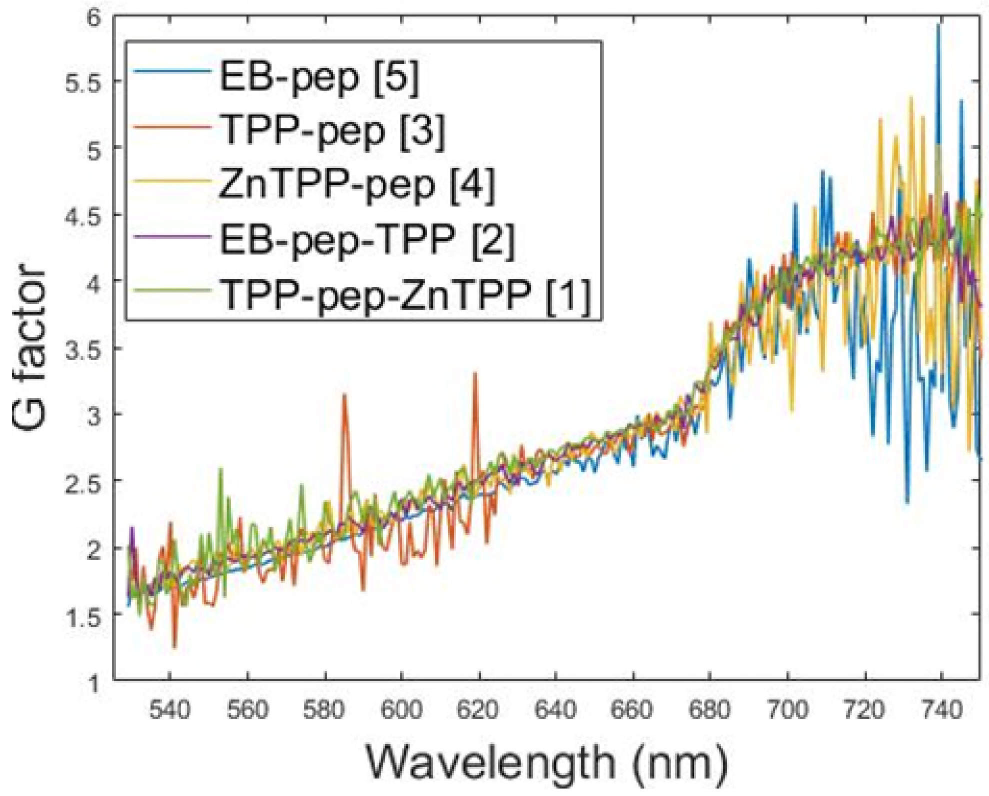

Figure S35. G-factor correction curve for steady-state fluorescence anisotropy plots for the five labelled peptide samples [**1**]-[**5**].

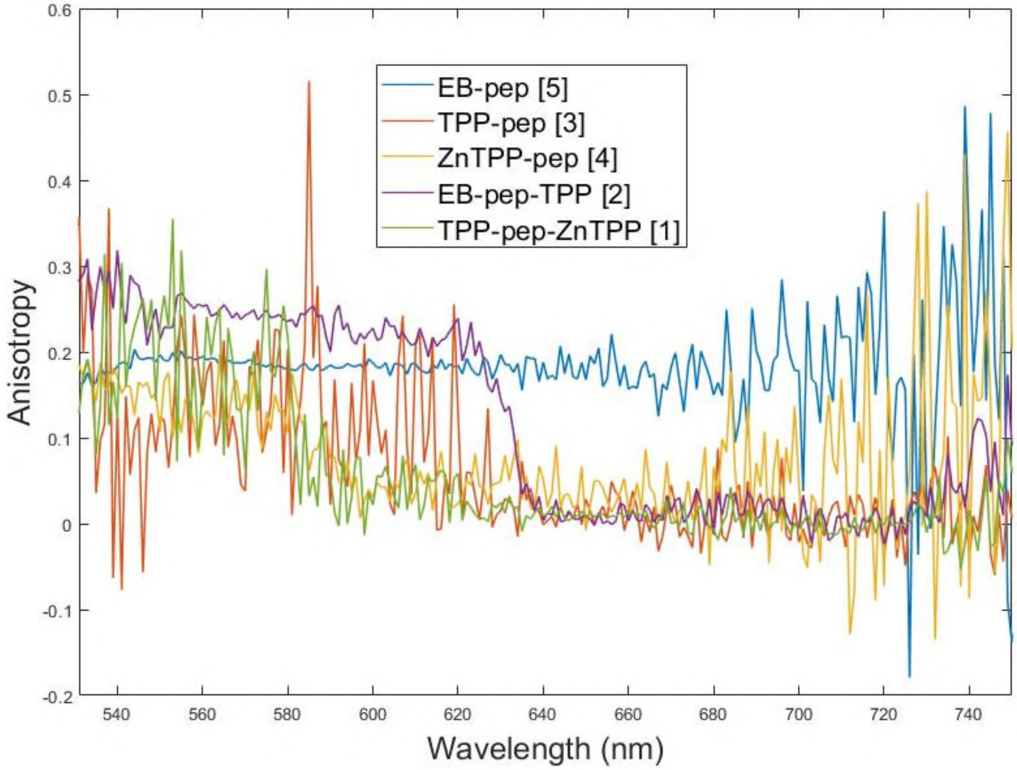

Figure S36. Steady-state fluorescence anisotropy plots for the five labelled peptide samples [1]-[5]. Within this plot the region of TPP emission is defined as 645-670 nm and 700-725nm, the region of ZnTPP emission as 580-630 nm and the region of EB emission as 540-590 nm.

Table S8. Average steady-state fluorescence anisotropy values calculated for systems [1]-[5] with the wavelength over which the average steady state anisotropies are calculated defined in brackets.

System	Average anisotropy (emission bandwidth defined in brackets)
EB-pep **[5]**	0.188 (540-590 nm)
TPP-pep **[3]**	0.008 (645-670 nm + 700-725 nm)
ZnTPP-pep **[4]**	0.058 (580-630 nm)
EB-pep-TPP **[2]**	0.246 (540-590 nm, EB region),<br>0.009 (645-670 nm + 700-725 nm, TPP region)
TPP-pep-ZnTPP **[1]**	0.045 (580-630 nm, ZnTPP region),<br>0.009 (645-670 nm + 700-725 nm, TPP region)

It has been reported that for systems with steady-state anisotropies (and r_c derived from time resolved measurements) lower than 0.2, one can assume (but not prove) sufficient rotational averaging occurs such that the $\kappa^2 = 2/3$ assumption for analysis of FRET data is appropriate, with a 5% uncertainty to the resultant R_0 values.[18] However other work provided different threshold values, such as a threshold value of $r_c = 0.22$, or a variation of ≤10% in $\Delta R_{app}(\kappa^2)$, the standard deviation of R_{app}, the inter-chromophore distance, calculated with $\kappa^2 = 2/3$ for a dye independent threshold of $\langle rc,\infty \rangle < 0.25$, where $\langle rc,\infty \rangle$ is the combined residual anisotropy.[19] As the steady state measurements, we conducted are a time averaged value we expect $r(\lambda em) > \langle rc,\infty \rangle$ and r_c.

The results of our steady-state anisotropy measurements indicate that system [1] and its single labelled analogues [3] and [4] lie below the 0.2 threshold and thus the $\kappa^2 = 2/3$ assumption is appropriate for this system and our results for this system are quoted with a 5% error bound on R_0 used in the calculation of the upper and lower distances calculated with different background corrections, see Section S2.8 for analysis of the FRET data.

The values for system [2] and the singly labelled analogues [3] and [5] show that the steady-state anisotropy in the region of the TPP emission is well below the 0.2 threshold while that in the region of the EB is 0.188 in the case of the single labelled peptide [5] and 0.246 in the case of the double labelled peptide [2]. These results indicate that the EB label is less mobile than the TPP label. As our values of r(λem) are <0.25, we have used a 10% error bound for our calculations of the distance in system [2], see Section S2.8 for analysis of the FRET data.

S2.8. FRET analysis

ZnTPP–TPP system

Excitation of molecule [1] at 510 nm primarily excites TPP and allows a FRET analysis comprising TPP as donor and ZnTPP as acceptor. Inspection of the spectral overlap (*J*) and the emission spectra of TPP and ZnTPP identified 561nm as the optimum wavelength to monitor FRET *via* quenching of the TPP emission (Fig. S37).

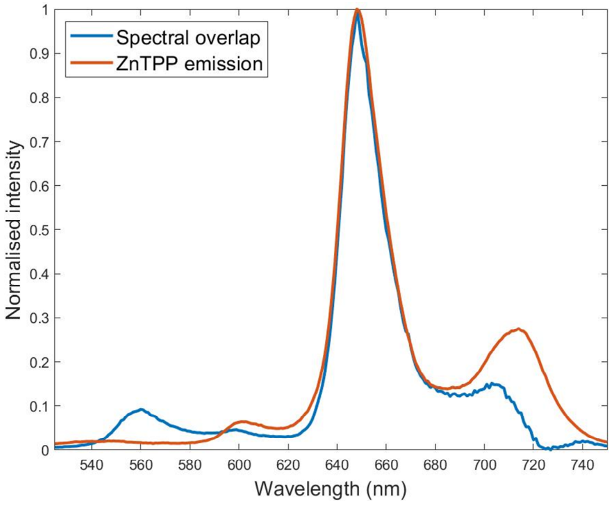

Figure S37. Spectral overlap (*J*) as compared to the emission spectrum of ZnTPP (acceptor) after excitation at 510 nm. Maximal overlap in the absence of acceptor emission intensity renders 561 nm as the optimal wavelength to carry out FRET analysis.

The FRET efficiency was calculated using Equation S1 with and without correction for the background signal, giving $E = 0.985$ and 0.864, respectively. This also provides a correction for any emission from the direct excitation of the ZnTPP. Using the values given in Table S9, r

values calculated from these E values are 21.6 Å and 31.8 Å, which we consider to be the primary lower and upper bounds to our estimate of the ZnTPP–TPP distance in [**1**]. We also included an uncertainty in the calculation of R_0 of 5%,[18] giving error bounds of r of 20.5 Å and 33.4 Å.

Excitation of molecule [**1**] at 567 nm primarily excites ZnTPP, which allows further analysis of this system using ZnTPP at the donor and TPP as the acceptor (Fig S38). However, comparison of the emission from [**1**], [**3**] and [**4**] showed problematic spectral overlap and significant enhancement of the donor (ZnTPP) emission upon excitation at this wavelength, which makes FRET calculations for this experiment inaccurate.

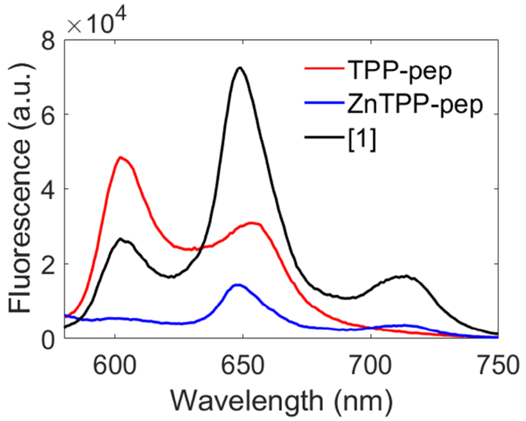

Figure S38. Fluorescence spectra of [**1**] (black) and the singly-labeled TPP-peptide [**3**] (red) and ZnTPP-peptide [**4**] (blue). Excitation was performed at 567 nm.

EB–TPP system

Excitation of molecule [**2**] at 510 nm allows a FRET analysis comprising EB as donor and TPP as acceptor due to the greater extinction coefficient of EB at 510 nm. Inspection of the spectral overlap (*J*) and the emission spectra of EB and TPP again identified 561 nm as the optimum wavelength to monitor FRET, *via* quenching of the EB emission (Fig. S39).

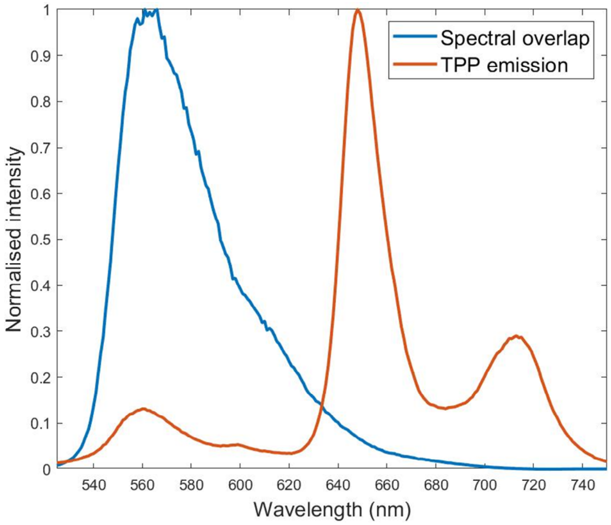

Figure S39. Spectral overlap (*J*) as compared to the emission spectrum of TPP (acceptor) after excitation at 510 nm. Maximal overlap in the absence of acceptor emission intensity renders 561 nm as the optimal wavelength to carry out FRET analysis.

The FRET efficiency was calculated using Equation S1 with and without correction for the background signal, giving $E = 0.913$ in both cases due to the negligible background in these measurements. We used a value of $\phi_{fl} = 0.095$ for EB, the r value was calculated from this E

value, giving $r = 26.3$ Å. Due to the larger anisotropy of this system we applied an error bound of ± 10% to this value,[19] giving a range of 25.0 Å to 27.6 Å for the EB–TPP distance in [**2**].

Table S9. Values of the variables used in the FRET calculations.

**Variable**	**Value used**
Refractive index (n) of ethanol[14]	1.4
Fluorescence quantum yield (ϕ_{fl}) of EB	0.095
Fluorescence quantum yield (ϕ_{fl}) of TPP	0.128
Molar extinction coefficient (ε) of TPP	451600 M^{-1} cm^{-1}
Molar extinction coefficient (ε) of ZnTPP	511800 M^{-1} cm^{-1}
Computed R_0 value for EB–TPP	38.85 Å ±1.95 Å
Computed R_0 value for ZnTPP–TPP	43.2 Å ± 2.16Å
Computed J value for EB–TPP	$2.5076x10^{15}$ $M^{-1}cm^{-1}nm^4$
Computed J value for ZnTPP–TPP	$3.3371x10^{15}$ $M^{-1}cm^{-1}nm^4$

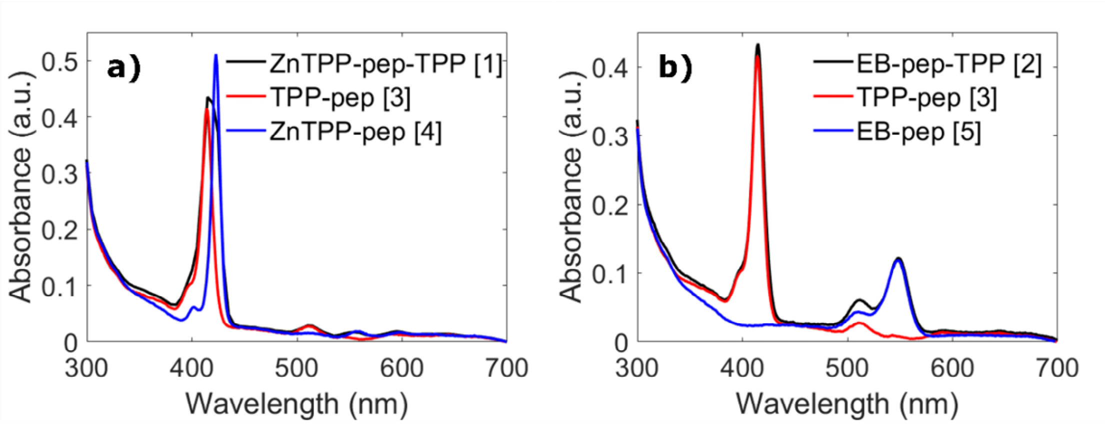

Figure S40. Room-temperature UV-Vis absorption spectra of the samples used in the FRET experiments. (a) Spectra of [**1**] (black), [**3**] (red) and [**4**] (blue), all at 0.7 μM. (b) Spectra of 1 μM [**2**] (black), 0.7 μM [**3**] (red) and 2 μM [**5**] (blue).

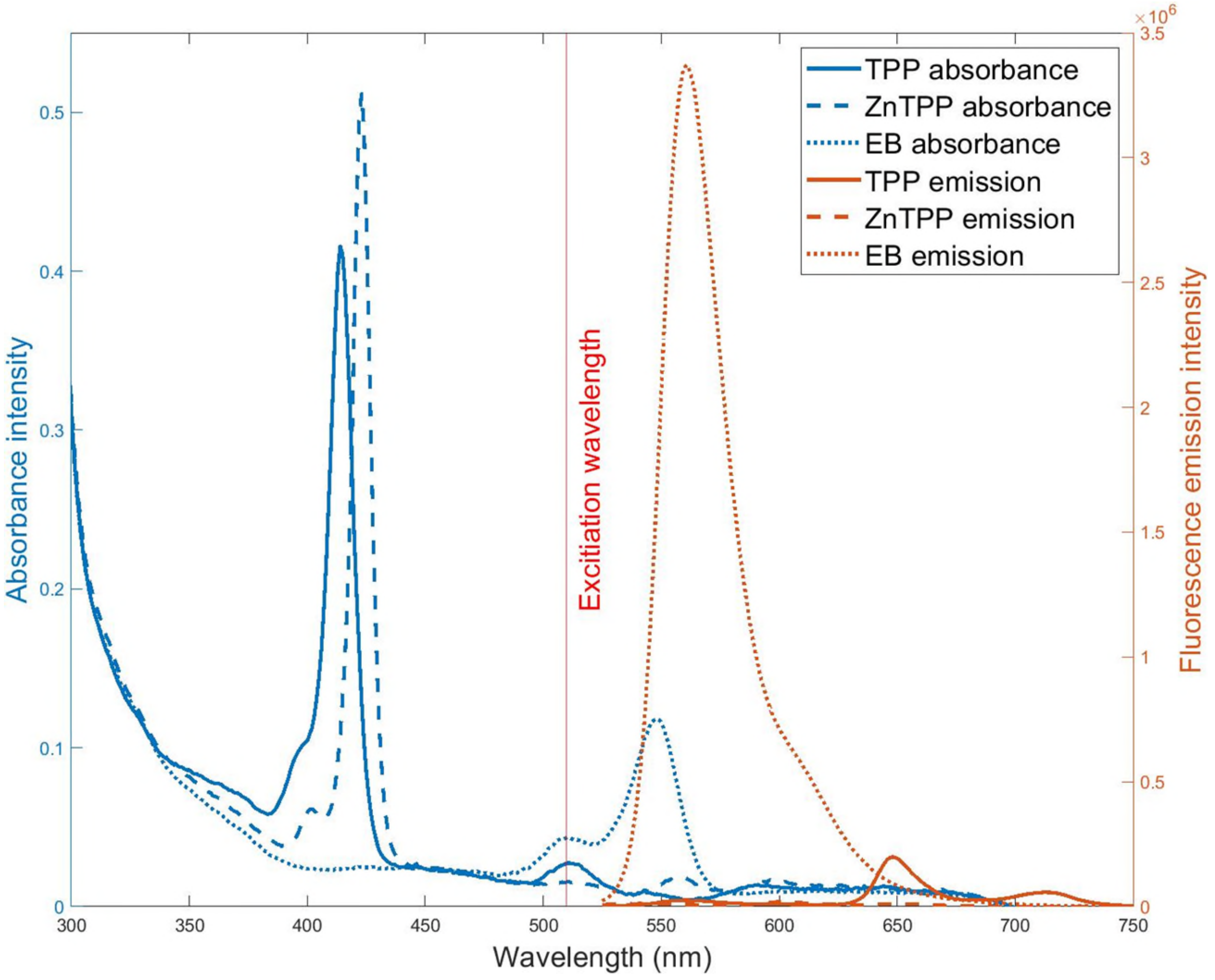

Figure S41. Absorbance (blue) and fluorescence emission (orange) spectra for each of the three chromophores used. Concentrations: 0.7 μM for [**3**] (TPP) and [**4**] (ZnTPP), 2 μM for [**5**] (EB). The excitation wavelength used for the FRET experiments (510 nm) is indicated.

REFERENCES

(1) Marafon, G.; Mosconi, D.; Mazzier, D.; Biondi, B.; De Zotti, M.; Moretto, A. Shaping Bioinspired Photo-Responsive Microstructures by the Light-Driven Modulation of Selective Interactions. *RSC Adv.* **2016**, *6* (77), 73650–73659. https://doi.org/10.1039/c6ra17673a.

(2) De Zotti, M.; Biondi, B.; Peggion, C.; Formaggio, F.; Park, Y.; Hahm, K. S.; Toniolo, C. Trichogin GA IV: A Versatile Template for the Synthesis of Novel Peptaibiotics. *Org. Biomol. Chem.* **2012**, *10* (6), 1285–1299. https://doi.org/10.1039/c1ob06178j.

(3) Bertran, A.; Henbest, K. B.; De Zotti, M.; Gobbo, M.; Timmel, C. R.; Di Valentin, M.; Bowen, A. M. Light-Induced Triplet–Triplet Electron Resonance Spectroscopy. *J. Phys. Chem. Lett.* **2021**, *12* (1), 80–85. https://doi.org/10.1021/acs.jpclett.0c02884.

(4) Stoll, S.; Schweiger, A. EasySpin, a Comprehensive Software Package for Spectral Simulation and Analysis in EPR. *J. Magn. Reson.* **2006**, *178* (1), 42–55. https://doi.org/10.1016/j.jmr.2005.08.013.

(5) Jeschke, G.; Chechik, V.; Ionita, P.; Godt, A.; Zimmermann, H.; Banham, J.; Timmel, C. R.; Hilger, D.; Jung, H. DeerAnalysis2006 - A Comprehensive Software Package for Analyzing Pulsed ELDOR Data. *Appl. Magn. Reson.* **2006**, *30* (3–4), 473–498. https://doi.org/10.1007/BF03166213.

(6) Sannikova, N. E.; Melnikov, A. R.; Veber, S. L.; Krumkacheva, O. A.; Fedin, M. V. Sensitivity Optimization in Pulse EPR Experiments with Photo-Labels by Multiple-Echo-Integrated Dynamical Decoupling. *Phys. Chem. Chem. Phys.* **2023**, *25* (17), 11971–11980. https://doi.org/10.1039/d3cp01056b.

(7) Worswick, S. G.; Spencer, J. A.; Jeschke, G.; Kuprov, I. Deep Neural Network Processing of DEER Data. *Sci. Adv.* **2018**, *4* (8), 1–18. https://doi.org/10.1126/sciadv.aat5218.

(8) Ibáñez, L. F.; Jeschke, G.; Stoll, S. DeerLab: A Comprehensive Software Package for Analyzing Dipolar Electron Paramagnetic Resonance Spectroscopy Data. *Magn. Reson.* **2020**, *1* (2), 209–224. https://doi.org/10.5194/mr-1-209-2020.

(9) Lakowicz, J. R. *Principles of Fluorescence Spectroscopy*, Third.; Springer: New York, 2006.

(10) Magde, D.; Wong, R.; Seybold, P. G. Fluorescence Quantum Yields and Their Relation to Lifetimes of Rhodamine 6G and Fluorescein in Nine Solvents: Improved Absolute Standards for Quantum Yields¶. *Photochem. Photobiol.* **2002**, *75* (4), 327. https://doi.org/10.1562/0031-8655(2002)075<0327:fqyatr>2.0.co;2.

(11) Taniguchi, M.; Lindsey, J. S.; Bocian, D. F.; Holten, D. Comprehensive Review of Photophysical Parameters (ε, Φf, Ts) of Tetraphenylporphyrin (H2TPP) and Zinc Tetraphenylporphyrin (ZnTPP) – Critical Benchmark Molecules in Photochemistry and Photosynthesis. *J. Photochem. Photobiol. C Photochem. Rev.* **2021**, *46*, 100401.

https://doi.org/10.1016/j.jphotochemrev.2020.100401.

(12) Thompson, M. *Thermodynamic and Kinetic Analysis of Bromodomain-Histone Interactions.*, 1st ed.; Elsevier Inc., 2009; Vol. 466. https://doi.org/10.1016/s0076-6879(09)66016-x.

(13) Pettersen, E. F.; Goddard, T. D.; Huang, C. C.; Couch, G. S.; Greenblatt, D. M.; Meng, E. C.; Ferrin, T. E. UCSF Chimera - A Visualization System for Exploratory Research and Analysis. *J. Comput. Chem.* **2004**, *25* (13), 1605–1612. https://doi.org/10.1002/jcc.20084.

(14) Frisch, M. J.; Trucks, G. W.; Schlegel, H. B.; Scuseria, G. E.; Robb, M. A.; Cheeseman, J. R.; Scalmani, G.; Barone, V.; Petersson, G. A.; Nakatsuji, H.; et al. Gaussian 09. Gaussian, Inc.: Wallingford CT 2009.

(15) Di Valentin, M.; Albertini, M.; Dal Farra, M. G.; Zurlo, E.; Orian, L.; Polimeno, A.; Gobbo, M.; Carbonera, D. Light-Induced Porphyrin-Based Spectroscopic Ruler for Nanometer Distance Measurements. *Chem. - A Eur. J.* **2016**, *22* (48), 17204–17214. https://doi.org/10.1002/chem.201603666.

(16) Williams, L.; Tischlik, S.; Scherer, A.; Fischer, J. W. A.; Drescher, M. Site-Directed Attachment of Photoexcitable Spin Labels for Light-Induced Pulsed Dipolar Spectroscopy. *Chem. Commun.* **2020**, *56*, 14669–14672. https://doi.org/10.1039/d0cc03101a.

(17) Murov, S. L.; Carmichael, I.; Hug, G. L. *Handbook of Photochemistry*, Second.; Marcel Dekker, Inc.: New York, 1993.

(18) Hellenkamp, B.; Schmid, S.; Doroshenko, O.; Opanasyuk, O.; Kühnemuth, R.; Rezaei Adariani, S.; Ambrose, B.; Aznauryan, M.; Barth, A.; Birkedal, V.; et al. Precision and Accuracy of Single-Molecule FRET Measurements—a Multi-Laboratory Benchmark Study. *Nat. Methods* **2018**, *15* (9), 669–676. https://doi.org/10.1038/s41592-018-0085-0.

(19) Agam, G.; Gebhardt, C.; Popara, M.; Mächtel, R.; Folz, J.; Ambrose, B.; Chamachi, N.; Chung, S. Y.; Craggs, T. D.; de Boer, M.; et al. Reliability and Accuracy of Single-Molecule FRET Studies for Characterization of Structural Dynamics and Distances in Proteins. *Nat. Methods* **2023**, *20* (4), 523–535. https://doi.org/10.1038/s41592-023-01807-0.
